# cAMP Control of HCN2 Channel Mg^2+^ Block Reveals Loose Coupling between the Cyclic Nucleotide-Gating Ring and the Pore

**DOI:** 10.1371/journal.pone.0101236

**Published:** 2014-07-01

**Authors:** Alex K. Lyashchenko, Kacy J. Redd, Peter A. Goldstein, Gareth R. Tibbs

**Affiliations:** 1 Department of Anesthesiology, Columbia University, New York, New York, United States of America; 2 Department of Neuroscience, Columbia University, New York, New York, United States of America; 3 Department of Anesthesiology, Weill Cornell Medical College, New York, New York, United States of America; Monell Chemical Senses Center, United States of America

## Abstract

Hyperpolarization-activated cyclic nucleotide-regulated HCN channels underlie the Na^+^-K^+^ permeable I_H_ pacemaker current. As with other voltage-gated members of the 6-transmembrane K_V_ channel superfamily, opening of HCN channels involves dilation of a helical bundle formed by the intracellular ends of S6 albeit this is promoted by inward, not outward, displacement of S4. Direct agonist binding to a ring of cyclic nucleotide-binding sites, one of which lies immediately distal to each S6 helix, imparts cAMP sensitivity to HCN channel opening. At depolarized potentials, HCN channels are further modulated by intracellular Mg^2+^ which blocks the open channel pore and blunts the inhibitory effect of outward K^+^ flux. Here, we show that cAMP binding to the gating ring enhances not only channel opening but also the kinetics of Mg^2+^ block. A combination of experimental and simulation studies demonstrates that agonist acceleration of block is mediated *via* acceleration of the blocking reaction itself rather than as a secondary consequence of the cAMP enhancement of channel opening. These results suggest that the activation status of the gating ring and the open state of the pore are not coupled in an obligate manner (as required by the often invoked Monod-Wyman-Changeux allosteric model) but couple more loosely (as envisioned in a modular model of protein activation). Importantly, the emergence of second messenger sensitivity of open channel rectification suggests that loose coupling may have an unexpected consequence: it may endow these erstwhile “slow” channels with an ability to exert voltage and ligand-modulated control over cellular excitability on the fastest of physiologically relevant time scales.

## Introduction

HCN channels represent the structural and functional fusion of two major branches of the potassium channel superfamily - depolarization-activated, K^+^-selective, K_V_ channels and the weakly voltage-sensitive, mono- and divalent cation permeable, cyclic nucleotide-gated CNG channels.

Gating in both HCN and depolarization-activated Kv channels involves stabilization of a dilated arrangement of their S6 helical bundles. In both channel classes this rearrangement is energetically coupled to motion of the four S1–S4 voltage-sensing domains and the concomitant reorientation of S4 positive charges with respect to the transmembrane field - albeit with an inverted coupling between the orientation of the sensors and opening of the gate [1], [2], [3], [4], [5], [6], [7], [8], [9], [10],[11],[12],[13],[14]. This contrasts with the weakly voltage sensitive CNG channels whose S4 is somewhat degenerate with respect to the canonical motif (with acidic residues often flanking a core that has a reduced number of positive charges [15]) and whose activation gate lies at the selectivity filter [16], [17], [18], [19], [20], [21], [22], [23], [24], [25] and not at the S6 bundle crossing that is dilated even in deactivated CNG channels [17], [22], [23], [26]. Similarly, while HCN channels display only a modest selectivity for K^+^ over Na^+^ (∼4: 1), not dissimilar to CNG channels, they contain a canonical K^+^-selective CIGYG motif at the selectivity filter rather than the degenerate filter of CNG channels wherein the tyrosine and a glycine are deleted [15], [27], [28], [29].

Unlike depolarization-activated Kv channels, opening of HCN and CNG channels is enhanced by agonist occupancy of cyclic nucleotide-binding domains (CNBDs). In each channel subunit, the CNBD is distally connected to the pore-lining S6 helix by an intervening motif, the C-linker [15], [27], [30], [31]. The architecture and motions of the cyclic nucleotide gating ring formed by the CNBDs and C-linkers appears to be well conserved between HCN and CNG channels [30], [32], [33], [34], [35], [36], [37], [38], [39]. Such conservation suggests that propagated changes that alter the pore of CNG channels may also be conserved and serve to alter the permeability properties of HCN channels, a hypothesis that has recently received support at least with respect to blocker binding sites in the inner vestibule of HCN2 [40].

Despite the many differences, fundamentally similar models are commonly used to describe activation and opening of all three classes of channels. Thus, while various forms of sequential models, each involving cooperative final opening transitions to a single open state, are the favored descriptions of Kv channel gating [41], [42], [43], [44], [45], such schemes are really only a strongly biased subset of the concerted allosteric models (based on the Monod Wyman and Changeux, MWC, formalism) that are commonly used to describe gating of HCN [30], [34], [35], [46], [47], [48], [49], [50], [51] and CNG channels ([15], [30], [33], [52], [53], [54] and see [39], [55], [56] for lucid discussions) – albeit the differences have important conceptual and functional consequences.

Evidence supporting the notion that the linkage between voltage sensors and the gate is weak in HCN and CNG channels is mounting [50], [56], [57], [58], [59]. Importantly, recent work on the architecture of the cyclic nucleotide gating ring has suggested that coupling of ring activation and channel opening (implied in MWC-type cyclic models) may not be as tight as previously believed. Structural and functional analysis of interactions within the gating ring indicate that various components of the gating ring can simultaneously adopt conformations attributed to activated and deactivated arrangements [30], [32], [60], [61]; analysis of channels composed of subunits that are competent and incompetent with respect to agonist binding indicate that the gating ring may operate as two functional dimers [33], [34] while patch clamp fluorimetry and isothermal titration calorimetry suggests inter-subunit cooperation maybe yet more complicated [62], [63]. Together, these findings suggest that the gating ring may adopt multiple functionally important and kinetically relevant arrangements. Such complexity is explicitly introduced by adoption of the modular model [30], [60] first used to describe gating of large conductance calcium-activated K^+^ channels [55].

It has previously been shown that intracellular Mg^2+^ acts as a voltage dependent blocker of open HCN channels [64], [65]. The voltage dependence of the block and its sensitivity to mutation of residues forming the inner face of the selectivity filter is consistent with the Mg^2+^ binding site lying close to or within the selectivity filter itself. Based on these observations we hypothesized that Mg^2+^ may act as a probe with which we could analyze the actions of cAMP on the architecture of the HCN channel pore.

Here, we show that agonist occupancy of the cyclic nucleotide gating ring does indeed modify the kinetics of Mg^2+^ block of HCN2 channels. Importantly, we show that the cAMP acceleration of block is unrelated to the enhancement of the channel's open probability (the kinetics of block and gating are so different that they are functionally decoupled) and that Mg^2+^ occupancy of the pore does not overtly alter the channel's closing reaction. Together, these findings show that the path to and from the ion's binding site discretely controls the microscopic kinetics of Mg^2+^ block with the clearest effect of cAMP being abolition of a slow component of the Mg^2+^ on-rate. These observations can be readily explained within a modular gating model by simply assuming that the different kinetics of Mg^2+^ block are a functional consequence of the architectural differences in the activated and deactivated conformations of the components of the nucleotide gating ring along with loose coupling between the ring and the pore. We hypothesize that a second messenger sensitivity of rectification represents a novel, and perhaps physiologically important, consequence of such plasticity in coupling.

## Materials and Methods

### Molecular Biology

1–50 ng of HCN2 or HCN2-R591E cRNA was prepared and injected into *Xenopus* oocytes as previously described [64].

### Electrophysiology

Excised inside-out patch clamp (IOPC) recordings were made from oocytes using an Axon Instruments Axopatch 200B amplifier (Foster City, CA) in the resistive mode with analogue compensation of linear ionic and capacitive currents applied. The 100 kHz output of the clamp's 4-pole Bessel filter was digitized at 200 kHz using an ITC-18 interface (Instrutech Corporation, Port Washington, NY) controlled by Pulse software (HEKA Elektronik, Lambrecht/Pfalz, Germany) without additional filtering. In all experiments, the extracellular solution was (mM) 112 KCl, 1 MgCl_2_, 1 CaCl_2_, 10 HEPES-free acid pH 7.4 (KOH). The intracellular solution was (mM) 112 KCl, 1 EGTA-free acid, 10 HEPES-free acid, pH 7.4 (KOH) that was supplemented with either 1 EDTA-free acid or MgCl_2_ at 0.3, 1, 2 or 3 mM and, where indicated, 30 or 300 µM cAMP (also added as the free acid). Throughout this manuscript, the concentrations of intracellular Mg^2+^ are discussed with respect to the added concentration but the appropriate free concentrations (0.276, 0.924, 1.859 and 2.804 mM as determined by MaxChelator: WebmaxC http://www.stanford.edu/%7Ecpatton/webmaxc/webmaxcE.htm) were used in all calculations.

Bath connections and sylgard-coated patch electrodes were as previously described (Lyashchenko and Tibbs, 2008). As currents tended to be large, especially at depolarized potentials, we routinely applied series resistance compensation with the resistance set equal to that of the electrode before seal formation (1–2 MΩ), the lag to 20 µs and the correction circuit to 95%. The seal resistance was typically 3–8 GΩ. Several lines of evidence indicate that uncompensated series resistance errors do not contribute to our descriptions of block. First, the time constant of block was independent of current amplitude across a >200-fold range (see File S1). Second, the conversion of block from mono- to bi-exponential behavior in the presence and absence of cAMP was independent of the current amplitude and persisted when Na^+^ replaced K^+^ as the main external charge carrier, conditions under which cAMP no longer enhanced the current amplitude (data not shown).

### Paradigms

Three types of voltage paradigm were used in these studies: A deactivation paradigm, a sequential IV paradigm and a depolarized conditioning envelope paradigm. In each case, the holding potential was −40 mV. Unless otherwise indicated, channels were activated by stepping to −155 mV for 2 s. After the activation step, the patch was stepped to: 1. +100 mV for 600 ms to follow both block and channel closing (the deactivation paradigm); 2. Potentials between −200 mV and +200 mV in 50 mV increments with test steps applied at 4 Hz (the sequential IV paradigm) or 3. +100 mV for various durations before returning to −155 mV for 2 s (the depolarized conditioning envelope paradigm). To ensure test steps were long enough to determine the time constant of block but short enough to prevent deactivation during the depolarizing epoch, the durations of the steps in the sequential IV protocol were increased from 1 ms at +200 mV to 3 ms at 0 mV in 0.5 ms increments then held at 3 ms for all negative potentials. To eliminate linear capacity and ionic currents not compensated by the analogue circuitry, in each protocol, we recorded leak records interlaced with the active records. To this end, patches were stepped from the holding potential to −155 mV for 5 ms before and after each test step during the sequential IV protocol, to −155 mV for 5 ms before and 150 ms after the step to +100 mV during the depolarized conditioning envelope protocol and −155 mV for 5 ms before the +100 mV tail step after which the voltages and durations were as in the cognate active sweep. Depending on the current amplitude, 2–17 leak sweeps were averaged and subtracted from the average of the corresponding, interlaced, 1–16 active sweeps. Throughout this manuscript, plateau tail current refers to that component of the tail that remains after the time required for development of block (irrespective of whether blocker is present or not) and before the onset channel of closing.

### Microscopic block kinetics

**Figure 1:**
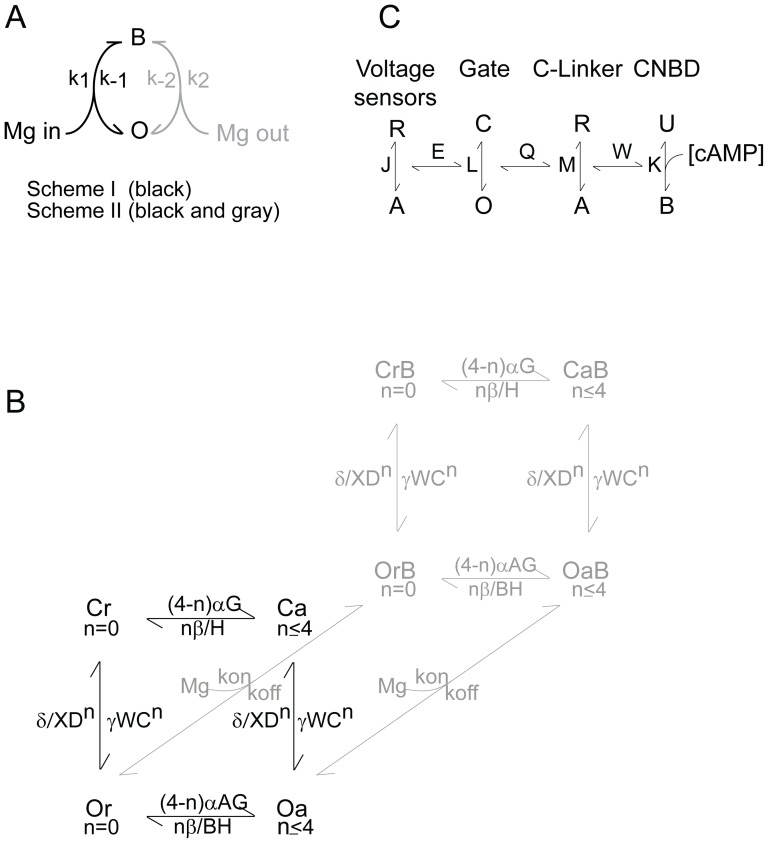
Schema and models describing HCN channel gating and Mg^2+^ block. **A.** Mg^2+^ block of HCN channels may occur *via* a simple bi-molecular process (Scheme 1) or *via* more complex processes (*e.g*., Scheme 2). For further details see Methods, Results and Discussion. **B.** Schematic representation of an allosteric gating reaction wherein Mg^2+^ can bind to and block the open channel (reactions going back into the plane of the page) but does so without altering the energetics of either activation (horizontal steps in the plane of the page) or opening (vertical steps). Further details of the model and the methods used to optimize the rate constants associated with gating are given in the methods section. **C.** Schematic representation of the modular model of gating. Here, as in the basic concerted model shown in panel B, voltage sensors can activate irrespective of the status of the pore and the pore can open whether the voltage sensors are activated or not but voltage sensor activation and pore opening results in a reciprocal stabilization when the allosteric coupling factor, E is >1. Furthermore, tighter binding of agonist when the gating ring is activated leads to a reciprocal stabilization of the ring and bound agonist if the allosteric factor W is >1. The critical divergence between the concerted and modular models is that in the latter case elements of the gating ring can be either activated or deactivated when the pore is open. As shown, pore opening is coupled to the status of the C-linker such that the open pore and the activated C-linker are reciprocally stabilized when the coupling factor Q is >1. Coupling between other modules is not excluded [55] but is not required for, nor included in, our simulations.

We have previously shown [64] that block of HCN channels by intracellular Mg^2+^ does not hew to the predictions of a simple impermeant block model (Fig. 1A, Scheme I). Specifically, neither the unblocked probability nor the apparent off rate decline exponentially ([64], see also below). The traditional, and simplest, explanation for the emergence of such anomalies accepts that the blocker is permeant (Fig. 1A, Scheme II - a model first proposed by Woodhull in 1973 [66]); however, Heginbotham and Kutluay have shown that such anomalies could arise as a consequence of changes in ion loading in a multi-ion pore with the blocker returning to the same compartment from which it arrived [67]. As the Woodhull scheme is better constrained, we use it as the framework for our quantitative analysis. A qualitative inspection shows the main conclusion of the study would not be altered were the Heginbotham and Kutluay model used instead (see Discussion).

In Scheme II all rates are exponentially described according to the general form shown in equation 1, the rate equation describing block is given by equation 2, the time constant of equation 2 is described by equation 3 and the probability that the channels are unblocked at equilibrium is given by equation 4 (see File S2 for derivations).
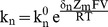
(1)

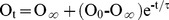
(2)




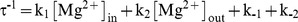
(3)




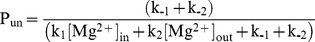
(4)


Here 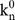 is the rate of the n^th^ step in the absence of an applied field; Z_m_ the valence of Mg^2+^; δ_n_ the effective electrical distance Mg^2+^ travels across the field (V) to reach the appropriate step's transition state assuming that all the effect of the field arises from a discrete effect on the Mg^2+^ ion. The sign of the exponent is negative for k_−1_ and k_2_ and positive for k_1_ and k_−2_. Throughout the manuscript, R, T and F have their usual meaning. O_0_, O_t_ and O_∞_ are, respectively, the occupancies of the unblocked open state at the onset of the test step, at time t after the beginning of that step and at an interval long enough for block to have equilibrated.

To determine the block time constant, we fit the pre-deactivation phase of the tail current with either equation 2 (with observed currents, I_0_, I_t_ and I_∞_ in place of occupancies) or a double exponential version thereof in the presence or absence of cAMP, respectively.

Equation 3 show that linear regression of plots of τ^−1^ versus [Mg^2+^]_in_ discretely yields k_1_ and that the ordinate intercept of such a regression analysis reports a compound rate constant k′′′  =  k_2_[Mg^2+^]_out_+k_−1_+k_−2_. Inspection of equation 4 shows that the product of the unblocked probability and τ^−1^ yields a different compound rate constant k′′ = k_−1_+k_−2_. While we cannot unequivocally determine the unblocked probability at any particular voltage (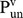), we know that it is directly proportional to current and, therefore, that the fraction of current remaining at t = ∞ with respect to that at t = 0 (at any particular test voltage) is equal to the ratio of unblocked probabilities (equation 5).
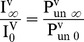
(5)


The unblocked current at equilibrium (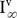) is determined from the asymptote of the exponential fit. 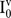 the instantaneous current at the onset of the blocking step, can be estimated one of two ways: 1) by extrapolation of the exponential fit of the decaying current to t = 0 or 2) by scaling the leak subtracted amplitude of the inward current immediately prior to the onset of the block step by the appropriate ratio of outward to inward currents derived from a block-free IV curve collected in the absence of intracellular divalent ions. We elected to use the scaling approach because this offered the better constrained measure, we indicate this by rewriting 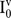 as 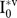. Similarly, 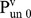 can be replaced with 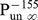 the unblocked probability at −155 mV. If we assume that this is close to 1, that is 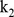 is small compared to k_−1_ at −155 mV, then we can use equations 3, 4 and 5 to obtain equation 6. That the IV relation is strongly inwardly rectifying in the presence of essentially symmetrical K^+^ and Mg^2+^ concentrations but essentially linear upon removal of internal Mg^2+^ supports the above assumption.
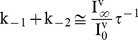
(6)


Evaluating the difference between the regression analysis compound rate constant (that includes k_2_[Mg^2+^]_out_) and the compound constant defined by equation 6 offers a way to estimate k_2_[Mg^2+^]_out_ at depolarized potentials.

Although block parameters will be best determined if both fast and slow components of block equilibrate before deactivation begins, optimizing the conditions to isolate the slow component (a high Mg^2+^ concentration at a strongly depolarized potential) will degrade measurement of the fast component (particularly of its amplitude) while lower concentrations and potentials will reduce the precision with which the slow component's time constant can be determined as it lengthens towards the duration of the pre-deactivation phase of the tail.

To monitor the extent to which extrapolation of the exponentials may compromise the estimate of relative amplitudes, we followed the ratio of the amplitude determined from extrapolation of the exponential fit function to 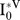 (as defined above). To explore the extent to which imperfect series resistance compensation may compromise quantification of block kinetics, we examined the correlation between the observed time constants (normalized with respect to the mean value at that Mg^2+^ concentration and voltage) and the current amplitude (as measured at −155 mV immediately prior to the depolarizing block step).

### Modeling and simulations of gating and block

To examine whether the effect of cAMP on Mg^2+^ blocking kinetics arose from a ligand-mediated change in the channel open probability and/or gating kinetics rather than through an effect of cAMP on block kinetics *per se*, we simulated activation and block in the presence and absence of cAMP using a 20-state model depicted schematically in Figure 1B. In this model, we assume that: 1.There are four identical and independent activation steps such that n represents the number of activated voltage sensors (and varies between 0 and 4), α and β represent the voltage dependent forward and reverse rate constants (defined by equations 7 and 8, respectively) and deactivated and activated states are depicted by subscripts r (resting) and a (activated); 2. Opening is a concerted reaction leading from either closed resting (Cr) or closed activated (Ca) states to open resting (Or) or open activated (Oa) states and γ and δ define the basal opening and closing rate constants; 3. A and B are variables that define how opening modifies the activation rate constants (α and β) while C and D are variables that define how activation modifies the rate constants underlying opening (γ and δ); 4. cAMP binding alters activation and opening if the variables W, X, G and H have non-unity values; 5. The K^+^ conductance of all unblocked open states (Or and Oa with n of 0 to 4) was assumed to be equivalent while the blocked open states (OrB and OaB with n of 0 to 4) were assumed to be non-conducting. As the opening and block/tail phases of the simulations were each at single potentials, we did not convert these values to currents as the conversion would not alter the shape of the traces while the amplitudes of the two phases do not carry any relevant information; 6. Mg^2+^ binding is inherently insensitive to the absence or presence of cAMP.
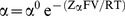
(7)




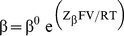
(8)




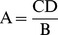
(9)




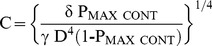
(10)




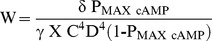
(11)


α^0^ and β^0^ are the forward and reverse rate constants for movement of the voltage sensors in the absence of an applied field; Z_α_ and Z_β_ are the charges associated with the forward and reverse motions of the voltage sensors. Conformation of the model to microscopic reversibility was achieved by defining the indicated parameters according to equations 9 to 11 during optimization.

We first optimized the activation and opening rate constants by simultaneously fitting the front plane of the model (no Mg^2+^ block) to open probability time courses in the absence and presence of a saturating concentration of cAMP using the Berkeley Madonna program. For these fits, we took HCN2 IOPC currents obtained in response to 10 s steps applied at 10 mV increments between −115 and −155 mV (in the absence of cAMP) −95 and −135 mV (in the presence of cAMP) and tail currents obtained at +100 mV in the absence and presence of cAMP. Inward currents were recorded in the presence of 1 mM internal Mg^2+^ while tail currents were recorded in the absence of intracellular Mg^2+^. These current records were converted into open probabilities as follows. Each sweep was: 1. Normalized to vary between 0 (channels closed) and 1 (maximal channel opening in that sweep); 2. Corrected to the appropriate fractional activation as determined from the Boltzmann equation wherein the V_1/2_ and slope were set equal to the observed values for these records (−125.6 and 3.3 mV in the absence of cAMP and −109.5 and 2.8 mV in the presence of the agonist); 3. Corrected to the activated open probability by multiplying the normalized corrected waves by 0.7 or 0.98 for data in the absence or presence of cAMP, respectively. These maximal open probabilities were obtained by non-stationary fluctuation analysis ([68], [69], [70]; data not shown). Note that normalizing the data in this manner assumes that deactivated opening is very unfavorable, a finding that is in keeping with prior observations [47], [70], [71]. As only a single tail voltage was used, error from that current was given a weight of 5 times that of the five activation sweeps. In the initial fitting cycles, we allowed both P_MAX_ and the deactivated opening equilibrium constant, L_0_, to vary. Although these parameters will clearly be constrained to be large and small, respectively (as a consequence of the data normalization we performed), their final fit determined values will, nonetheless, be strongly influenced by the gating kinetics at intermediate potentials. The values listed in Table 1 are those that appeared to allow for the best solutions from such time course fitting. In one series of fits, cAMP was assumed to only act on the opening isomerization (G and H were constrained to 1) while in a second series of fits G and H were also allowed to vary. The gating charges associated with the forward and reverse reactions were allowed to vary but were constrained such that they were equivalent in the absence and presence of cAMP.

**Table 1:** Optimized values of rate constants and gating modifier variables used in the simulations shown in Figure 8.

	Opening and activation		Opening only		
	-cAMP	+cAMP	-cAMP	+cAMP	
α^0^	1.6×10^−7^		2.2×10^−7^		s^−1^
Z_α_	3.0		3.4		
β^0^	2.7		4.4		s^−1^
Z_β_	0.5		0.6		
γ	0.003		0.002		s^−1^
δ	1400		864		s^−1^
A	46.7 (#^9^)		23.9 (#^9^)		
B	0.7		1.3		
C	4.9 (#^10^)		4.8 (#^10^)		
D	7		6.3		
W	1	120 (#^11^)	1	151 (#^11^)	
X	1	3.7	1	7.0	
G	1	1.3	1	1	
H	1	0.6	1	1	
.				1.064×10^6^	M^−1^ s^−1^
δ_1_		0.164		0.164	
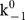 .				4346	s^−1^
δ-_1_		0.306		0.306	
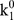 . 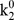 .				0	M^−1^ s^−1.^
δ_2_		—		—	
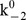 .				21	s^−1^
δ_−2_		0.303		0.303	
I_∞_/I_0_		0.046		0.046	

Gating parameters were estimated using time course fitting of HCN2 currents while those describing Mg^2+^ block kinetics were derived from block in the presence of cAMP as shown in figures 4 and 5 (see methods for details). Where plus or minus cAMP parameter windows are left blank, the values are constrained to be equivalent to that shown in the other condition for that model. Superscripted #'s refer to the appropriate equations in the methods that were used to determine the value of the indicated parameter.

We then asked whether the cAMP-dependence of gating could account for the appearance of a cAMP-dependence of block. To do so, we first set both gating and block parameters in our 20-state model (Fig. 1B) to those determined in the presence of nucleotide (where k_ON_ equals k_1_[Mg^2+^]_in_ and k_OFF_ the sum of k_−1_ and k_−2_ with k_1_, k_−1_ and k_−2_ being exponentially-distributed with respect to voltage, as *per* equation 1, see Results for detailed description of parameter determination). We then adjusted the gating parameters, and only the gating parameters, to their control values and examined whether this change altered block.

To examine how an explicit cAMP-sensitivity of Mg^2+^ block may emerge, we simulated the gating behavior of HCN2 channels using the modular model developed by Horrigan and Aldrich [55] and Craven and Zagotta [30], [60]. This simplified expansion of the four dimensional modular model (which does not account for the tetrameric nature of the channels) can be envisioned as two nested cubes wherein horizontal transitions represent movement of the voltage sensors, vertical transitions represent the opening isomerization and movements from the front plane to the back represent C-linker activation. Connections between the corners of the inner and outer cubes represent cyclic nucleotide binding.

In this model (see Fig. 1C), J, L, M and K represent the equilibrium constants for activation of the voltage sensors (as per equation 12 where J^0^ is the equilibrium constant for voltage sensor activation in the absence of an applied field and Z_J_ is the gating charge moved by the sensors), the opening of the pore, activation of the C-linker and nucleotide binding to the CNBD while E, Q and W represent the allosteric coupling factors linking these equilibria. Values of factors were: J^0^ = 1.1×10^−12^, Z_J_ = 5.3, L = 3×10^−6^, M = 2.3×10^−4^, K = 1.9×10^6^, E = 2.3×10^5^, Q = 1×10^4^, W = 60. In equations 12–15, G = 1+K[cAMP], H = 1+WK[cAMP], X = G+MH, Y = G+MQH.
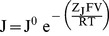
(12)




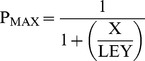
(13)




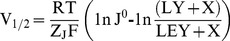
(14)




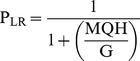
(15)


Equations 13 to 15 define how P_MAX_, V_1/2_ and P_LR_ (the probability that the C-linker of an open channel is in its resting conformation) vary as a function of the cAMP concentration, [cAMP]. As this model describes the uncoupling of the activation status of the linker and the pore, we assign fast versus slow block to the status of the linker but the model remains valid even if another component of the modal machinery is the actual determinant of the barrier to the Mg^2+^ binding site.

### Fitting and statistical analysis

Data analysis was performed in PulseFit (HEKA Elektronik) or with user generated functions in IgorPro (Wavemetrics Corporation, Lake Oswego, OR). SigmaStat V3.1 (Systat Software, Point Richmond, CA) was used to perform Student's t-tests (differences between two populations) and one-way ANOVA with post hoc Holm-Sidak analysis (comparison of multiple populations). A P <0.05 was considered significant. Data are presented as mean ± SEM except for quotients of means which are reported with respect to their 95% confidence interval.

### Reagents

Electrophysiology reagents were of the highest purity from Sigma.

## Results

### cAMP-accelerates intracellular Mg^2+^ block of open HCN2 channels

**Figure 2:**
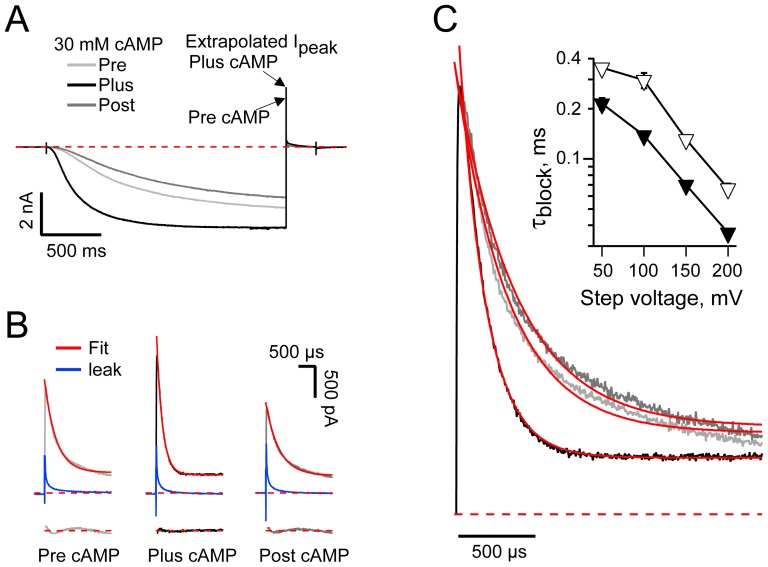
cAMP accelerates [Mg^2+^]_in_ block of HCN2 channels. **A.** HCN2 channels activated at −155 mV and deactivated at +100 mV in the presence of 2 mM intracellular Mg^2+^ and the absence (Pre), presence (Plus) and following washout (Post) of 30 µM cAMP. Arrows indicate the instantaneous tail current amplitudes in the absence and presence of cAMP (determined by zero time extrapolation of fits of a single exponential function – *e.g*,. as shown in **B** and **C**). Records are active sweeps before subtraction of flanking leak sweeps acquired using the deactivation protocol (see Methods). **B.** Expanded views of the initial 2 ms of the +100 mV tails from **A** following subtraction of the averaged interlaced leak sweeps (shown in blue). Solid red lines represent fits of a single exponential function. The residuals from the fits are shown vertically offset for clarity. In this and all other figures, dashed red lines represent the zero current level. **C.** Current records (and exponential fits thereunto) normalized to the observed peak amplitude of the plus 30 µM cAMP tail current. Inset: the time constants of decay of the initial phase of the HCN2 tail currents in the presence of 2 mM Mg^2+^ and the absence (open symbols) or presence (closed symbols) of 30 µM cAMP (10 to 27 determinations per point) are significantly different at each potential (Student's t-tests). Data acquired from deactivation and sequential IV protocols (see Methods) were pooled in this plot.

Figure 2A shows representative HCN2 currents recorded at −155 mV in the absence, presence and following washout of 30 µM cAMP. Inspection shows that the presence of the ligand reversibly accelerated channel activation and enhanced the amplitude of the inward current. These observations are consistent with cAMP acting to enhance a rate limiting voltage-independent opening reaction [47] and stabilize a relatively unfavorable opening equilibrium (data not shown; see also [47], [57], [60]).

Figure 2B shows expanded views of the early phase of the tail currents from the records in Figure 2A. The initial time course of the tails is, as previously reported [64], [65], dominated by a voltage-dependent block of the outward current by intracellular Mg^2+^. Surprisingly, inclusion of 30 µM cAMP appears to reversibly accelerate the block. This impression is reinforced by comparison of the records after scaling each record to the maximal current amplitude observed in the presence of the nucleotide (Fig. 2C). To quantify the effect of cAMP on the Mg^2+^ block kinetics, we fit the initial phase of the tail currents with a single exponential function (solid red lines superimposed on the data in Figs. 2B and C). A plot of the time constants of block as a function of the depolarizing step potential shows that cAMP doubled the rate of the blocking reaction at all potentials (Fig. 2C inset).

### cAMP-acceleration of intracellular Mg^2+^ block is mediated via ligand occupancy of the cyclic nucleotide gating ring

Are the effects of cAMP on block due to activation of the gating ring or does this arise from a non-specific effect of the ligand (such as the introduction of a low concentration of a contaminating cation that has a high affinity for the Mg^2+^ site)? To address this question, we performed two tests. First, we determined the effect of 30 µM cAMP on HCN2-R591E, an HCN2 channel wherein a critical arginine residue in the cyclic nucleotide-binding domain has been replaced with a glutamate rendering the channel insensitive to µM levels of cAMP while leaving basal gating unaltered [34], [72]. Second, we compared the effects of 30 µM cAMP with those of 300 µM cAMP on the block kinetics in wild type HCN2 (note that both concentrations of agonist exceed that required to saturate the CNBD as reported by the effect of ligand on channel gating, data not shown and [34], [72]). If the effect of cAMP addition is mediated *via* a contaminating particle, we would predict that the block kinetics of HCN2-R591E will be as sensitive to 30 µM cAMP as are the block kinetics of HCN2 while a 10-fold increase in cAMP concentration should result in an equivalent further acceleration of the block kinetics of HCN2. To explore these questions we used the sequential IV voltage paradigm as that allowed us to isolate the block kinetics at a series of test potentials while simultaneously monitoring the activation and deactivation kinetics at potentials where block was less marked (see Methods for details).

**Figure 3:**
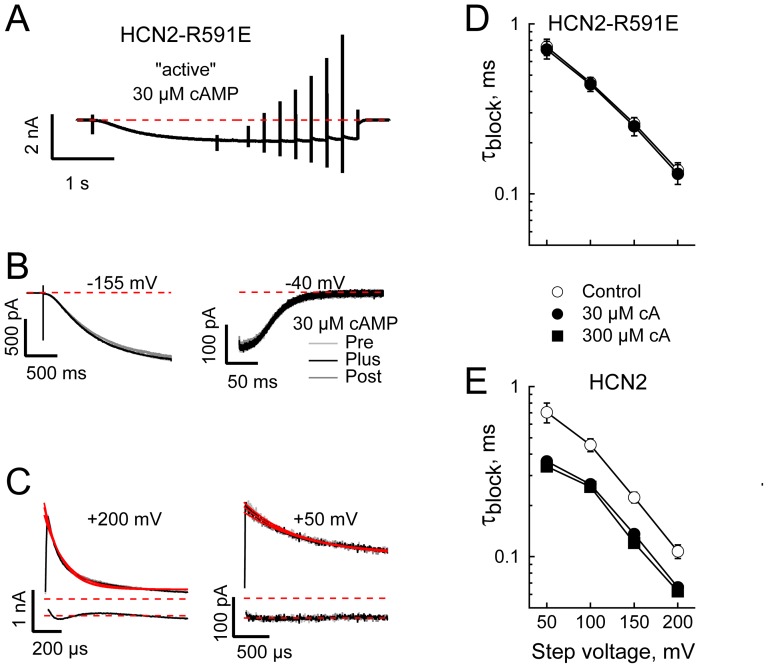
cAMP acceleration of [Mg^2+^]_in_ block is mediated *via* ligand occupancy of the cyclic nucleotide-gating ring. **A.** Average of 8 consecutive active sweeps acquired from a patch expressing HCN2-R591E channels in response to the sequential IV voltage paradigm. Intracellular Mg^2+^ was 1 mM. **B.** Expanded view of activation (Left panel) and deactivation (at the holding potential of −40 mV; Right Panel) of HCN2-R591E obtained in the absence (Pre), presence (Plus) and following washout (Post) of 30 µM cAMP. Records are from same patch as A and are each averages of 8 sweeps acquired in response to the active paradigm before subtraction of the averaged interlaced leak records. **C.** Expanded views of the leak subtracted currents recorded at +50 and +200 mV (as indicated) in the absence, presence and following washout of 30 µM cAMP (traces and legend as in **B**). Red lines are fits of a single exponential function. Residuals are shown vertically offset for clarity. **D,E.** Time constant of block by 1 mM intracellular Mg^2+^ of HCN2-R591E (**D**) and HCN2 (**E**) in the absence or presence of 30 or 300 µM cAMP. For HCN2 but not the cAMP-disabled construct, HCN2-R591E, block kinetics in the presence of cAMP were significantly different from block in the absence of cAMP while the speed of block of HCN2-R591E in the absence or presence of cAMP was not different from that of block of HCN2 in the absence of the nucleotide (one-way ANOVA at each voltage with 11–20 determinations per point).

Figure 3A–C show data from a representative recording obtained with HCN2-R591E in the presence of 1 mM intracellular Mg^2+^ in the absence, presence and following washout of 30 µM cAMP. As anticipated, the presence of cAMP did not alter either activation or deactivation kinetics (see expanded and superimposed views of the opening time courses at −155 mV and the closing reaction at −40 mV - left and right panels of Figure 3B, respectively). Importantly, superimposed views of currents recorded at +50 mV (Fig. 3C right) and +200 mV (Fig. 3C left) indicate that block of HCN2-R591E was also insensitive to the presence or absence of cAMP.

The mean single exponential time constants of block determined at varying potentials in the presence of 1 mM intracellular Mg^2+^ for HCN2-R591E and HCN2 are shown in Figure 3D and E, respectively. In agreement with the data presented in Figure 2, cAMP accelerated Mg^2+^ block in HCN2 (time constants observed in both 30 or 300 µM cAMP were significantly different from control at all voltages). Importantly however, the kinetics of block of HCN2 in the presence of 300 µM cAMP were indistinguishable from those observed in the presence of 30 µM nucleotide while the kinetics of block of HCN2-R591E were not affected by the presence or absence of cAMP and were not different to the block kinetics of HCN2 in the absence of ligand (comparisons were by one-way ANOVA at each voltage). These data reveal that cAMP acts to modify block *via* its association with the CNBD. It is interesting to note that that these data also act to support the contention that mutation of the conserved arginine in the CNBD does not perturb the overall architecture of HCN channels.

### In the absence of cAMP, Mg^2+^ block is biphasic

**Figure 4:**
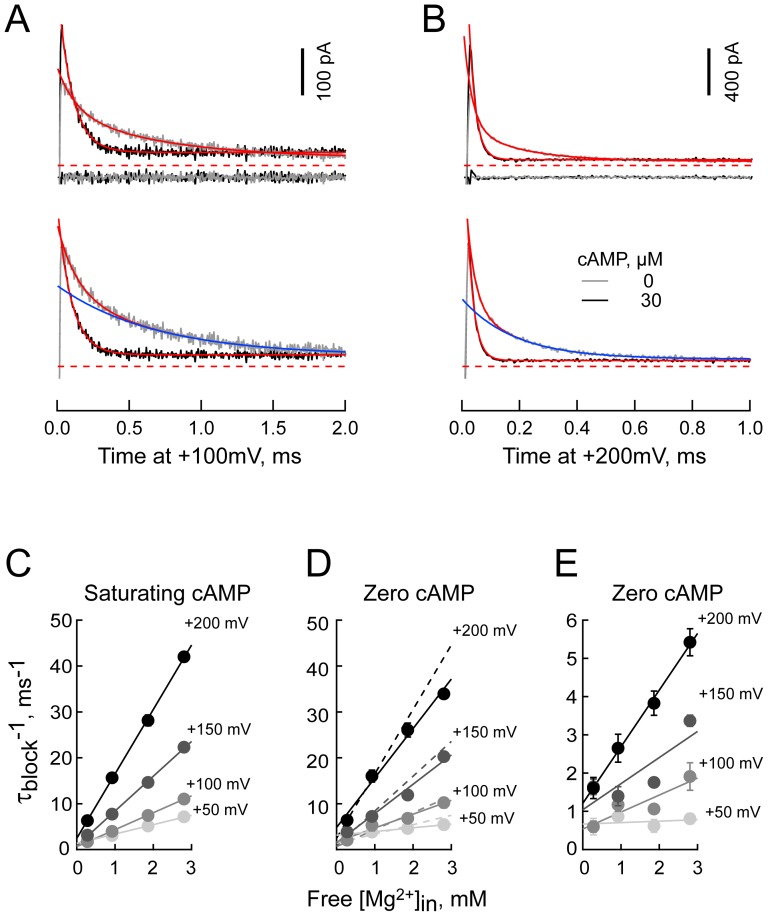
Bi-exponential behavior of [Mg^2+^]_in_ block in the absence of cAMP. **A,B.** Expanded views of leak subtracted currents recorded at +100 (A) and +200 mV (**B**) in the presence of 3 mM Mg^2+^ and absence (gray) or presence (black) of 30 µM cAMP before (upper) and after (lower) normalization to the observed peak tail current. Red lines are fits of single or double exponential functions (30 and 0 µM cAMP, respectively). Blue lines represent the slow component of the double exponential fits. Residuals are shown offset below the current records in the upper panels. Data acquired with the sequential IV protocol (see Methods). **C–E.** Plot of 1/τ_BLOCK_ versus voltage at the indicated Mg^2+^ concentrations in the presence (**C**) and absence (**D,E**) of cAMP. **D** and **E** plot the data for the fast and slow phases of block in the absence of cAMP, respectively. The dashed lines in **D** are the fit lines from **C**. r^2^ values for fits to 50, 100, 150 and 200 mV data are **C**: 0.9974, 0.9997, 0.9991 and 0.9997; **D**: 0.9985, 0.9676, 0.9782 and 0.9854; **E**: 0.1004, 0.8165, 0.7023 and 0.9966. Data are from 7–26 and 7–27 separate patches for plus and minus cAMP, respectively.

Figure 4A and B show representative HCN2 pre-deactivation tail currents obtained in the presence of 3 mM Mg^2+^ and absence and presence of 30 µM cAMP at +100 (Fig. 4A) and +200 mV (Fig. 4B). In keeping with the data presented above, the rapidly decaying component of the HCN2 tail current is monophasic in the presence, but not the absence, of cAMP. In the absence of nucleotide, the early component is well fit by a bi-exponential function.

We can consider three simple explanations for this behavior: 1. The blocking mechanism in the absence and presence of cAMP is different; 2. The blocking mechanism in the absence and presence of cAMP is equivalent but, due to a mass action effect of closed channels, a slow second component appears when the open probability is significantly less than unity; 3. In the absence of nucleotide, there are two slowly interconverting populations of channels each of which block according to a similar reaction but do so with different kinetics. Below, we present evidence that the third interpretation is correct.

### Analysis of microscopic Mg^2+^ blocking kinetics suggests cAMP occupancy of the CNBD eliminates a slow blocking configuration of HCN2 channels

**Figure 5:**
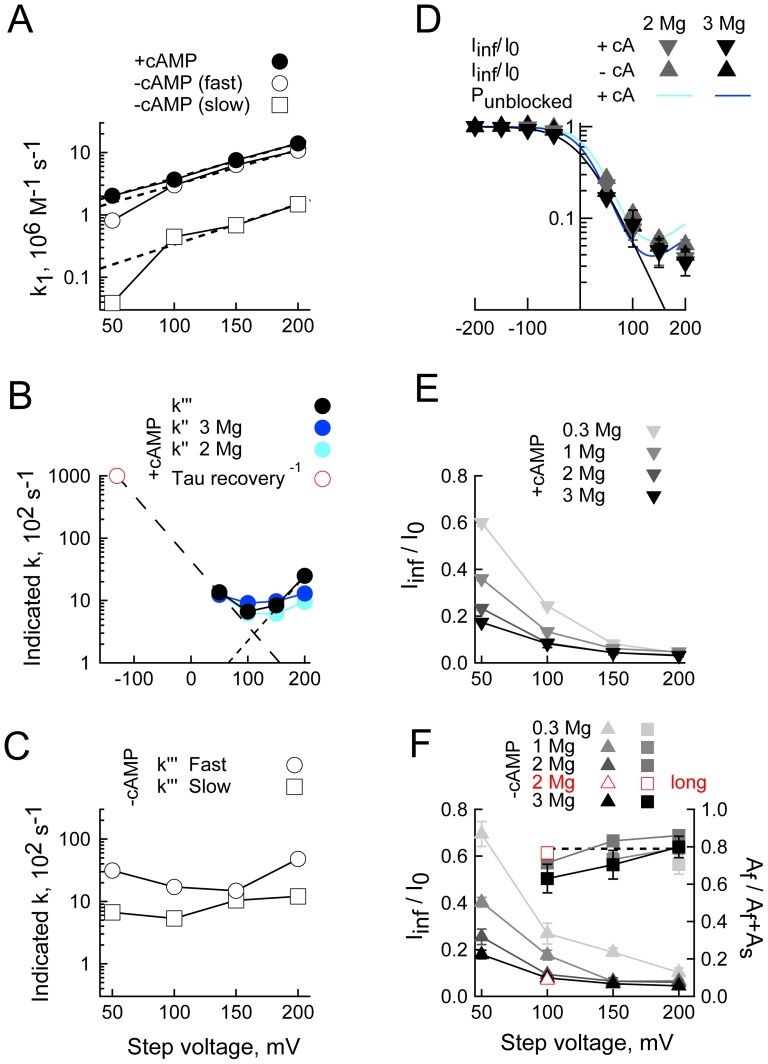
cAMP abolishes a slow blocking population of channels. **A.** k_1_ determined from the slopes of the regression lines in Figure 4C–E plotted against the depolarizing step potential in the presence or absence of cAMP. Dashed lines represent fits of equation 1 (see text for details). **B,C.** Compound rate constants determined in the presence (**B**) and absence (**C**) of cAMP. Black symbols: k′′′ (equal to k_2_[Mg^2+^]_out_+k_−1_+k_−2_ at 1 mM [Mg^2+^]_out_) as obtained from the y-intercepts in Figure 4C–E. Teal and blue symbols: k′′ (equal to k_−1_+k_−2_ obtained according to equation 6) at 2 mM and 3 mM [Mg^2+^]_in_, respectively. The red symbol at −135 mV is set to 10^5^ s^−1^ in keeping with the observation that recovery of current is faster than the time constant of the clamp at that voltage [64]. The long and short dashed lines (**B**) represent the optimized behavior of k_−1_ and k_−2_, respectively obtained from fits to the black and red circles. This fit reported 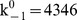 s^−1^, δ-_1_  = 0.306, 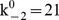 s^−1^ and δ_−2_  = 0.303. **D.** Black and grey symbols show the fractional unblocked current. The ratios at 0 mV are omitted as this potential is close to the reversal potential and, therefore, poorly defined. Teal and blue lines: the probability channels are unblocked (equation 4) using the scheme II parameters determined in **A–C**. The black line is a fit of equation 4 wherein both k_2_ and k_−2_ are zero; it represents the predicted exponential behavior if Mg^2+^ block were to accord to Scheme I. **E,F.** Plots of the fractional unblocked current and the relative amplitude of the fast component of block (zero time extrapolation of the fast component with respect to the sum of zero time amplitudes of the fast and slow components, A_f_ and A_s_ respectively – right hand aspect of **F**). Open red symbols (**F**) represent the estimates obtained in the presence of 2 mM Mg^2+^ and absence of cAMP when a block window of 10 ms was employed in place of the normal 2 ms window. The dashed line (**F**) is the mean of the fractional fast amplitude determined in the presence of 0.3, 1, 2 and 3 mM Mg^2+^ at 200 mV.

In Figure 4C–E, the inverse time constants describing current decay due to development of block (single exponential in the presence of cAMP but double exponential in the nucleotide's absence) are plotted as a function of [Mg^2+^]_in_. Figure 5A plots the slopes of the regression lines in Figure 4C–E; within Scheme II, these data report k_1_ as a function of voltage (see equation 3). We obtained estimates of 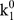 and δ_1_ of 1.064 ×10^6^ M^−1^ s^−1^ and 0.164 in the presence of AMP and 0.822×10^6^ M^−1^ s^−1^ and 0.165 and 7.6×10^4^ M^−1^ s^−1^ and 0.189 for the fast and slow components observed in the absence of agonist. Figure 5B and C report the ordinate intercepts (zero [Mg^2+^]_in_) of the regression lines in Figure 4C–E; within Scheme II these data report the compound rate constant k′′′ which is equal to k_2_[Mg^2+^]_out_+k_−1_+k_−2_ as per equation 3. Figure 5B additionally reports k′′ (obtained by equation 6 and, within Scheme II, approximately equal to k_−1_+k_−2_) as well as the inverse time constant of relief of block at −155 mV. The difference between k′′′ and k′′ defines the maximum allowed value of the external Mg^2+^ on rate (at 1 mM [Mg^2+^]_out_) at each voltage. As the difference is the smallest at lower voltages (where k_2_ should be at its largest) the data show this term contributes little, if at all to the observed block behavior across the depolarized domain. Accordingly, k_2_ was assumed to be zero at depolarized potentials and <<k_−1_ at hyperpolarized potentials. Note that k′′ cannot be determined in the absence of cAMP as it is not possible to unequivocally parse P_UN_ between the fast and slow components. The behavior of equilibrium block is plotted in Figures 5D–F. Several things are apparent from these data.

First, in the presence of cAMP, block closely hews to the predictions of the single site permeant block model depicted in Scheme II wherein external Mg^2+^ has very poor access to the blocking site. Thus: 1) The on rate of block is linearly related to [Mg^2+^]_in_ (Fig. 4C); 2) When it moves from the cytoplasm to its transition state, Mg^2+^ experiences ∼0.16 of the field (as derived from the slope of the lines in Figure 5A), suggesting the transition site is within the pore but towards the inner face of the vestibule; 3) The compound rate constants k′′′ and k′′ both display a concave relationship with voltage and are essentially identical. This identity is inconsistent with an alternative hypothesis, that residual current represents incomplete block of K^+^ flux through Mg^2+^ occupied channels; such flux would result in gross overestimation of k′′ but would not affect k′′′. As k_2_ decreases with increasing voltage, the correspondence between k′′ and k′′′ allows us to further conclude that k_2_[Mg^2+^]_out_ is effectively zero in the depolarized domain. As such, we set k_2_ to zero in fitting and modeling routines. The modest deviation between estimates of k′′ and k′′′ at +200 mV does not contradict these conclusions; at +200 mV the contribution of k_2_ should be at its smallest not its largest; 4) An estimation of P_UN_ from the fitted values of the rate constants of Scheme II coincides closely with the observed value of the fraction of current that remains unblocked (Fig. 5D), demonstrating that the parameter estimates for the model account well for all aspects of the observed block behavior.

Second, the fast component of block in the absence of cAMP has very similar properties to block in the presence of cAMP. Thus, k_1_ for the two conditions essentially superimpose (Fig. 5A – open versus filled circles). This observation accords with the correspondence in the τ^−1^ plots in Figure 4C and D. While we cannot discretely probe the external on rate of the fast component by evaluating k′′′ minus k′′ (k′′ cannot be determined in the absence of cAMP as it is not possible to unequivocally parse P_UN_ between the fast and slow components) the close correspondence in terms of τ^−1^ and k_1_ suggests fast block in the absence of cAMP is essentially identical to block in the presence of nucleotide.

Third, the clearest effect of removing cAMP is to generate a slow phase of block that has an approximately 10-fold lower on-rate but which has an essentially unaltered voltage dependence (Fig. 5A open squares). Although less well determined, Figure 5C suggests that k′′′ for the slow component of block in the absence of cAMP is also slowed; the validity of this interpretation is reinforced by the fact that we see no clear difference in equilibrium block behavior in the presence and absence of cAMP (Fig. 5D). It is noteworthy though that, as predicted by a single site model such as Scheme II, the intracellular Mg^2+^ on rate of this component (k_1_[Mg^2+^]_in_) is still linearly dependent on [Mg^2+^]_in_ at least at the higher potentials (Fig. 4E). We attribute the increased scatter at lower potentials and [Mg^2+^]_in_ to inaccuracies arising from the brevity of the window available to determine the block kinetics.

Fourth, while the permeant block model of Woodhull can account for all aspects of block in the presence of cAMP, the presence of the additional, extracellular, route for Mg^2+^ entry and egress is, alone, insufficient to account for bi-exponential block in the absence of cAMP; inspection of equations 2 and 3 show that at any particular set of Mg^2+^ concentrations and voltage block will be inherently single exponential. This demonstrates that an additional behavior of the channels must impinge on the blocking mechanism in the absence of nucleotide.

### Relative amplitudes of the fast and slow blocking populations in the absence of cAMP

Figures 5E and F plot the fractional unblocked current and the relative contribution of the fast component of block as a function of both step potential and Mg^2+^ concentration in the presence and absence of cAMP, respectively. The relative amplitudes in Figure 5F are plotted for only those voltages and Mg^2+^ concentrations where fits reliably settled to the fully blocked current level. At voltages where block appears to be fully developed (the fractional unblocked current settles to a value of ∼5%), the relative amplitude of the slow component in the absence of cAMP is estimated to be ∼20% (right hand plot of Fig. 5F). However, the latter value may be an underestimate. Thus, at the higher potentials and Mg^2+^ concentrations, extrapolation of exponential fits to the tail current as block develops (e.g. Figs. 2C and 4A and B) appears to overestimate the instantaneous amplitude (1.42±0.06, 1.52±0.06, 1.37±0.05, and 1.46±0.17 for 3, 2, 1 and 0.3 mM Mg^2+^ at 200 mV, respectively – see methods for details) and this overestimation will tend to predominantly reflect an error in extrapolation of the fast component of block. To test whether the blocking reaction in the absence of cAMP is near equilibrium at 2 ms, we extended the block window to 10 ms. Figure 5F shows lengthening the block window did not alter the parameter estimates.

### cAMP-acceleration of intracellular Mg^2+^ block is mediated via ligand control of block kinetics and not via cAMP-sensitive changes in channel open probability

Above, we analyzed the data assuming that slow block in the absence of cAMP represented Mg^2+^ association with a separate population of channels. An alternative interpretation of the data (albeit one that would not be without physiological relevance) is that cAMP alters block kinetics as a secondary consequence of its ability to increase the channel open probability and slow channel closing. Indeed, while we have previously shown that in the presence of cAMP the recovery of current upon return to negative potentials is essentially instantaneous [64], inspection of the Figure 3A shows a sag in the HCN2-R591E current during the inter-pulse intervals in the sequential IV paradigm. Not only was such a reopening phase seen with HCN2-R591E in the absence or presence of cAMP, it was also apparent with HCN2 in the absence of cAMP (data not shown). Such an observation could be evidence that closing in such recordings is sufficiently fast that it contaminates the block records at depolarized potentials.

Here, we use both experimental and modeling approaches to demonstrate that 1. Block and opening are kinetically decoupled such that modulation of these two processes by cAMP represents effectively independent mechanisms of control of channel function and 2. The origin of the observed sag lies in the cyclic nature of HCN2 channel gating reactions (including expansions of the basic 10-state gating scheme shown in Figure 1B to incorporate a modal behavior of the voltage-sensors; [50], [51], [73]) and not an overlap of gating and blocking kinetics.

To address the first point we asked if 1. The absence or presence of intracellular Mg^2+^ altered the maximal closing rate of HCN2 channels (a rate that has been shown to be independent of voltage at very depolarized potentials and is, thus, expected to be insensitive to the effect of an altered Mg^2+^ concentration acting *via* a change in the surface potential); 2. cAMP alters closing kinetics within the window when block develops; 3. A decrease in open probability through manipulation of the activation step introduces a slow component of block in the presence of cAMP and 4. Block, deactivation and closing could be decoupled within a kinetically realistic model of HCN2.

**Figure 6:**
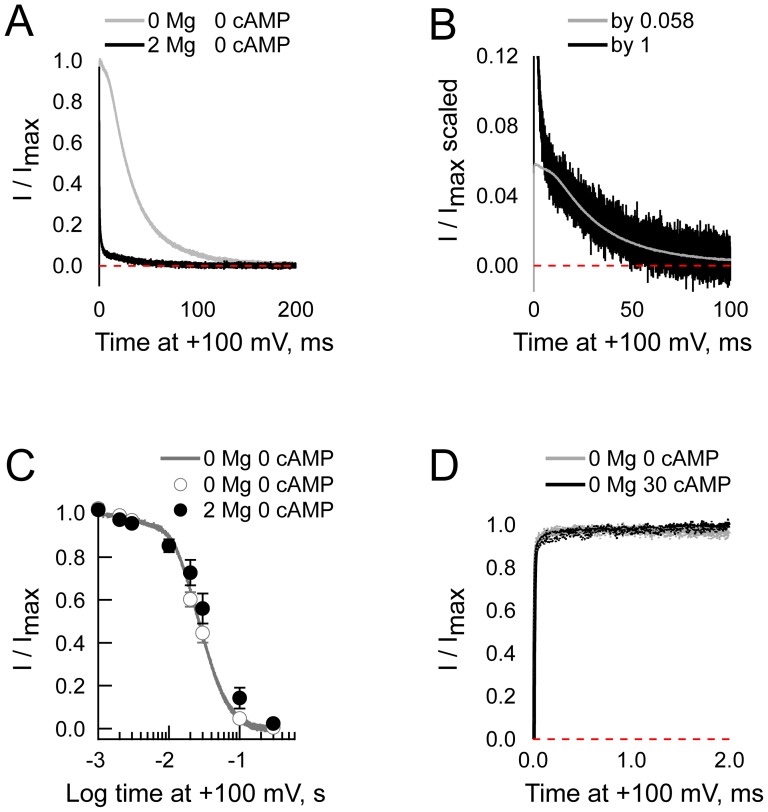
[Mg^2+^]_in_ does not modify closing kinetics and closing does not intrude into the block time domain. **A,B.** Leak sweep subtracted tail currents in the absence or presence of 2^2+^ and absence of cAMP normalized to the peak amplitude of each recording then averaged (**A**: 10 and 16 separate recordings) or same records after scaling of the 0 Mg^2+^ record (**B**). The SEM of these averaged records is included as a pixilated halo around the records in **A** and **D**. **C.** Deactivation envelopes determined in the absence (open circles) and presence (filled circles) of 2 mM Mg^2+^ (3–8 determinations per point). The continuous line represents a mean +100 mV tail current (14 separate recordings each normalized to the peak amplitude before averaging). At no time were the envelope amplitudes in the absence and presence of Mg^2+^ significantly different (Student's t-tests). **D.** The initial 2 ms of +100 mV tail currents collected in the absence of internal Mg^2+^ and the absence or presence of cAMP (normalized to the peak amplitude during the 2 ms window then averaged).

Figure 6A shows normalized mean tail currents recorded at +100 mV in the absence and presence of 2 mM Mg^2+^. This comparison shows the potent effect the alkaline earth metal has on the current carrying capacity of HCN2 channels at depolarized potentials but does not permit the effect of the divalent cation on the voltage independent deactivation kinetics to be readily considered. To explore this, we performed further tests. First, we scaled the mean normalized tail current obtained from recordings in the absence of Mg^2+^ and superimposed this scaled trace on the slow phase of the tail current recorded in the presence of Mg^2+^ (Fig. 6B). Second, we compared the deactivation time course in the presence of Mg^2+^ (observed by determination of the instantaneous tail envelope at −155 mV; representative recordings obtained with our deactivation envelope paradigm are shown below) with that in the absence of Mg^2+^ (observed by following either the instantaneous tail envelope at −155 mV or the continuous tail current at +100 mV; Fig. 6C). These data show that while the presence of Mg^2+^ serves to blunt the amplitude of the tail it does not overtly alter the rate of channel closure. Note also that the factor by which the Mg^2+^-free tail current is scaled to superimpose on the residual deactivating current observed in the presence of Mg^2+^, 0.058, is very similar to the estimate of the residual current determined in analysis of the microscopic kinetics of block above (Fig. 5). These findings indicate that the closing phase in the presence of Mg^2+^ discretely represents closure of channels that are at steady state with respect to Mg^2+^ occupancy and that interaction of Mg^2+^ with the pore does not alter the energy of closure of the gate. Such insensitivity of deactivation gating to Mg^2+^ occupancy allows us to use the kinetics of deactivation in the absence of Mg^2+^ to ask whether closing in the absence of cAMP is likely to affect the observation of block.

Figure 6D overlays normalized mean tail currents recorded at +100 mV in the absence of internal divalent cations and the absence or presence of a saturating concentration of cAMP. These data show that during the initial 2 ms window there is no marked change in the HCN2 channel open probability in either the absence or presence of cAMP. This conclusion is consistent with the results of our modeling studies (see below).

**Figure 7:**
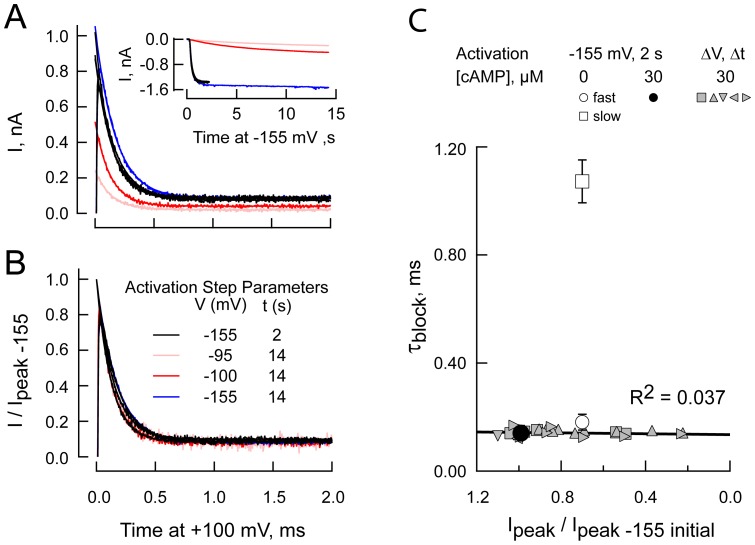
Slow block is controlled by cAMP occupancy of the gating ring and not open probability. **A.** +100 mV leak sweep subtracted pre-deactivation HCN2 tail currents observed immediately following activation (see inset for opening trajectories) at −155 mV for 2 s (black traces) or 14 s (blue) or for 14 s at either −100 mV (red) or −95 mV (pink) in the presence of 2 mM intracellular Mg^2+^ and 30 µM cAMP. Superimposed smooth lines are fits of each trace with a single exponential function. The V_1/2_ and slope factor determined from a fit of the Boltzmann function to an activation curve constructed from 10 s sweeps were −105.7 and 4.5 mV, respectively (data not shown). **B.** Sweeps and fits from the block records shown in **A** each normalized to the instantaneous amplitude determined from the cognate exponential fit. **C.** Single exponential time constants of block from five patches such as that shown in **A–B** (gray shaded symbols). These are compared to the mean (± SEM) time constants of block by 2 mM Mg^2+^ following activation at −155 mV for 2 s in the presence (filled circle, n = 22) or absence (open symbols, n = 27) of cAMP (open circle and square: fast and slow components of a two exponential fit). Submaximal activation voltages varied between −95 and −115 mV (in 5 mV increments) while times varied between 5, 8 or 14 s at the submaximal voltages and between 2 or 14 s at −155 mV. For clarity, and because varying the durations and activation voltages had no effect on block kinetics (other than altering the open probability at the onset of the block epoch - see, for example, **A** and **B**), we do not differentiate between short and long activation pulses or the various activation potentials in this plot.

We next asked whether lowering the open probability alone would mimic the effect of removing cAMP. Figure 7A shows the block phase of HCN2 tail currents obtained in the presence of cAMP following channel activation at −155 mV and at potentials that elicit submaximal activation (see inset for the corresponding inward currents). Figure 7B shows the block phase of the tail currents each normalized to their instantaneous tail current amplitude. Inspection suggests that block following activation at submaximal voltages was qualitatively indistinguishable from that observed upon activation at −155 mV. To examine this quantitatively, we fit these records (and similar data from four other patches) with a single exponential function and plotted the recovered time constants as a function of the amplitude of the instantaneous tail current relative to the amplitude of the instantaneous tail current obtained with the initial −155 mV, 2 s sweeps - a surrogate measure of the open probability. Figure 7C shows that the time constant of block is invariant when the open probability is changed. These results confirm that activation of the gating ring controls block kinetics directly and not via a cAMP-sensitivity of the open probability.

**Figure 8:**
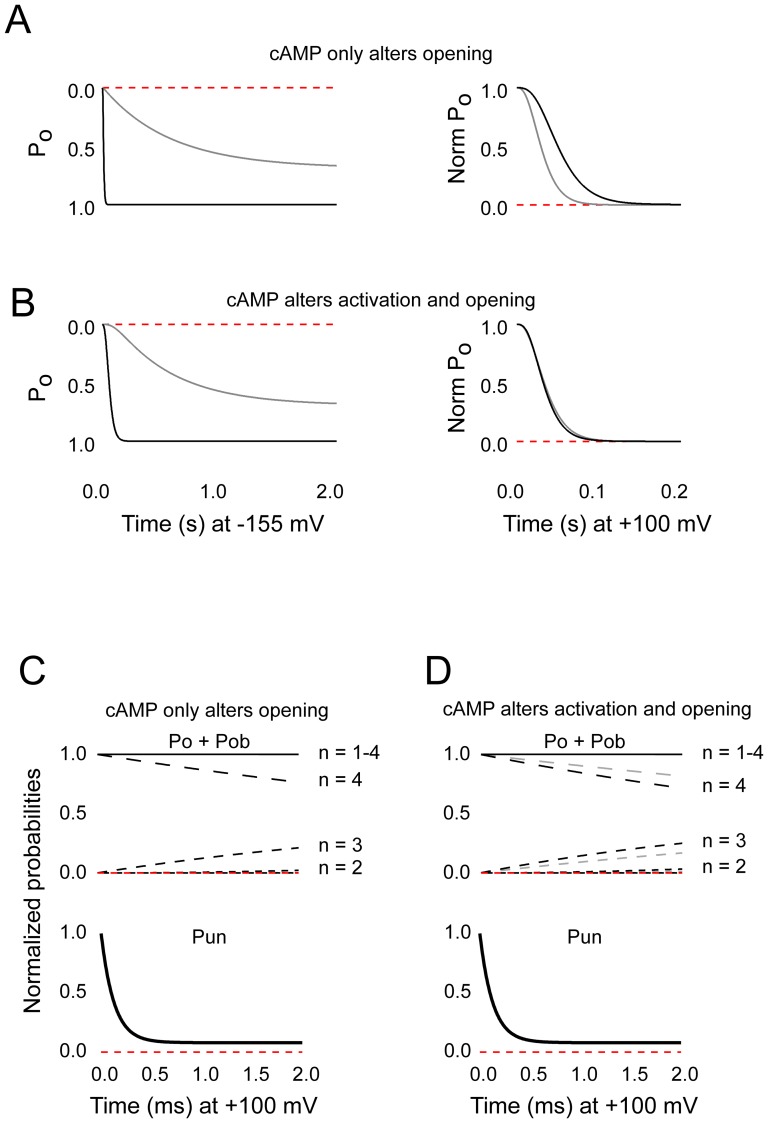
cAMP control of [Mg^2+^]_in_ block and channel opening are kinetically decoupled processes. **A,B.** Simulated HCN2 currents at −155 mV (Left) and +100 mV (Right) in the absence (Gray) and presence (Black) of cAMP and the absence of intracellular Mg^2+^. The current records were simulated using the rate constants shown in Table 1 wherein cAMP did (B) or did not (A) alter activation transitions. **C,D.** Probability of occupancy of sum of open and open blocked states with the indicated number of activated voltage sensors (upper panels) and open unblocked probability (lower panels) when cAMP alters only the opening isomerization (**C**) or both activation and opening reactions (**D**). In all panels, the probabilities were normalized to the initial maximal open probability under the specified conditions to simplify comparison of simulations generated in the presence and absence of cAMP. Note that the plus and minus cAMP traces in the lower panels superimpose.

The implication of the above findings is that the kinetics of activation and opening are so different to those of block that the two processes are effectively decoupled. To consider this explicitly, we developed a 20-state kinetic model (Fig. 1B) wherein k_ON_ and k_OFF_ (see Methods) were set to the values describing block in the presence of cAMP and the gating parameters varied between those that describe gating in the presence of cAMP to those that describe gating in the absence of cAMP (see Table 1 for values). The resulting simulations (Fig. 8) show two important features. First, as observed experimentally, channels did not close during the initial 2 ms at +100 mV (solid black lines in the upper families of traces in Figures 8C and D) irrespective of whether the effect of cAMP on gating was restricted to the opening isomerization (Fig. 8C) or was allowed to partition between that reaction and the activation transitions (Fig. 8D). Second, the normalized block time course (lower families of traces in Figures 8C and D) was indeed insensitive to the cAMP-mediated changes in the much slower gating reactions (plus and minus cAMP traces in the lower families of Figure 8C and D superimpose).

**Figure 9:**
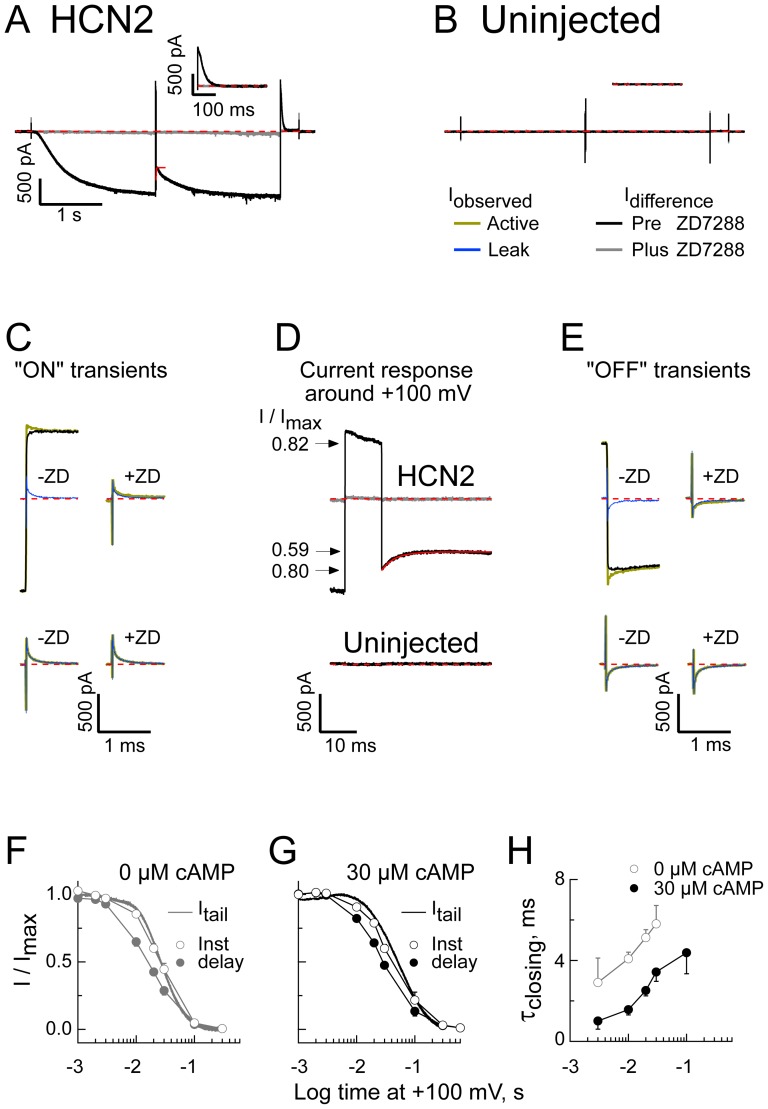
Inter-pulse sag arises from cAMP-dependent anomalous closure at hyperpolarized potentials, not closure at +100 mV. **A,B.** Averaged active records obtained in response to the depolarized conditioning envelope paradigm (wherein the +100 mV sojourn was 10 ms) from a cell expressing HCN2 (**A**) or an un-injected cell from the same donor frog (**B**) before (Black) and after (Gray) inclusion of 300 µM ZD7288 in the bath. In each case, the inset shows the tail currents at +100 mV obtained after the second −155 mV epoch. **C–E.** Records obtained before (**C**), during (**D**) and after (**E**) the conditioning 10 ms step to +100 mV from the HCN2 (Upper traces) and un-injected (Lower traces) recordings shown in **A** and **B**, respectively. Yellow and blue traces are the averaged active and leak records. Where included, the black and gray traces are the difference currents obtained before (Black) and after (Gray) inclusion of 300 µM ZD7288. **F,G.** Leak sweep subtracted continuous +100 mV tail currents (normalized to the peak amplitude of each recording then averaged; 14 and 13 separate recordings in the absence and presence of cAMP, respectively) and the normalized amplitudes of the instantaneous (Inst) and delayed (delay) envelope currents upon return to −155 mV following steps of varying duration to +100 mV (3–11 determinations per point) are each plotted with respect to time at +100 mV. The instantaneous and delayed amplitudes were determined from fits of a single exponential (*e.g*., red line in **A** and **D**) and plotted as a function of the current amplitude at −155 mV immediately prior to the +100 mV conditioning step. **H.** Time constant of the −155 mV closing phase as a function of preceding +100 mV conditioning interval.

Finally, we examined the origin of the inter-pulse sag present in sequential IV recordings in the absence of cAMP activation of the gating ring (Fig. 3A). To explore this question, we used the depolarized conditioning envelope paradigm (see Methods). Figure 9A and B show representative recordings obtained from a cell expressing HCN2 (Fig. 9A) or from an un-injected cell from the same donor frog (Fig. 9B) when the +100 mV conditioning step was 10 ms long and both Mg^2+^ and cAMP were absent from the bath solution. Although this record suggests that ∼40% of the channels have closed during the 10 ms at +100 mV, consideration of the channel's behavior immediately before, during and after the brief step to +100 mV indicates this is not the case.

Figure 9C and E show the active, leak and net (upper records only) currents observed during the initial phase of the step to +100 mV and during the return to −155 mV (“On” and “Off” transients, respectively) for recordings from the un-injected cell (lower records) and the HCN2 expressing cell (upper records). Figure 9D shows the leak-subtracted net currents observed before, during and after the conditioning step to +100 mV in the absence (black) and following inclusion of the HCN selective inhibitor ZD7288 (gray). Note first that this approach faithfully isolates the HCN2 current. Thus, subtraction of the leak from the active traces in the recording from the un-injected cell revealed no ZD7288-sensitive current component (in the lower records in Figure 9D the black and gray traces are flat and superimpose around zero current) whereas the current obtained from the HCN2 patch showed a robust asymmetric current (black trace in the upper family of traces in Figure 9D) that was completely eliminated by ZD7288 (gray trace). Importantly, we see that the 10 ms +100 mV step closes only ∼18–20%, not 40%, of the channels as measured at either the tail potential (where I_MAX_ and I are the amplitudes at the beginning and end of the +100 mV step) or at −155 mV (where I_MAX_ is the current before the step to +100 mV and I is the current immediately upon return to −155 mV). Rather, much of the closing occurred over the subsequent 10–20 ms with I/I_MAX_ at −155 mV declining to 0.59. Figure 9F and G plot the −155 mV instantaneous and delayed I/I_MAX_ ratios with reference to channel closing at +100 mV (monitored by the continuous tail currents, I_tail_) while Figure 9H plots the apparent time constant of the anomalous −155 mV closing phase (in the absence and presence of cAMP as indicated). Note that similar results were observed when Mg^2+^ was present in the internal solution (data not shown).

These experiments reveal that the sag observed in Figure 3A (when the depolarizing window is much shorter) is due to channels that close after the block has developed and not from closing that is proceeding synchronously with block. Parenthetically, while such hysteresis does not uniquely define the pattern of connectivity, it is most consistent with models wherein open and closed states communicate irrespective of the activation status of the gating apparatus (see the Introduction and Discussion for further consideration) and its amplitude and kinetic properties will aid in constraining a more general gating model of HCN channels.

### Residual current at depolarized potentials is carried primarily by K^+^ and not Mg^2+^

It has been suggested that the HCN pore is divalent ion permeable [74], [75], [76] and that this may represent a chemical signaling role for these channels in addition to their conventional role as electrical transducers. To add to this debate, we next sought to use our data to quantify the maximum contribution that Mg^2+^ can make as a charge carrier.

Given a single channel conductance of 2.4 pS and an essentially linear single channel IV (in the absence of [Mg^2+^]_in_ block - see File S3); a reversal potential of ∼0 mV (which is as expected and confirmed experimentally); and a residual current of ∼5% of the unblocked value (see Figs. 4, 5 and 6 and associated text), we calculate that the time averaged residual single channel current is 24 fA at +200 mV. At 200 mV, the compound rate constant k′′′ is ∼2500 s^−1^ (at 1 mM [Mg^2+^]_out_; Fig. 5B). If we assume that at this potential this is solely due to k_−2_ (an outward Mg^2+^ flux), this would represent a charge transfer of 5000 elementary charges per second or 0.8 fA. That is, the maximal Mg^2+^ transfer rate can account for no more than 3.3% of the observed residual current. This calculation suggests the bulk of the residual current is carried by K^+^ transiting channels that are temporarily unblocked and that divalent ion transfer through the HCN pore is paltry (at least with respect to Mg^2+^) at best.

## Discussion

We have investigated the coupling between the cyclic nucleotide gating ring and the permeation path of HCN2 channels. We have examined this by analyzing the kinetics of Mg^2+^ block. In the absence of cAMP, Mg^2+^ block has both fast and slow components; the presence of cAMP eliminates slow block independently of the nucleotide's effect on gating. Importantly, the slow block is associated with a slowing of both the Mg^2+^ on-rate and the metal's off rate (albeit the latter is better observed by the lack of effect of gating ring activation on Mg^2+^ equilibrium binding than in the relatively poorly defined off rate *per se*) with no marked difference in the voltage dependencies. The simplest interpretation of these observations is that the energy barriers Mg^2+^ experiences in transit to and from its binding site at the selectivity filter is controlled by the activation status of the gating ring; the barriers are higher when the ring is deactivated and lower when the ring is activated. Kinetic control of blocker binding with no discernible effect on the Mg^2+^ binding site *per se* accords with the observation that Mg^2+^ occupancy does not alter cAMP association energetics [40].

### cAMP-sensitive bi-exponential Mg^2+^ block – Is interpretation of this as Mg^2+^ binding to two channel populations reasonable?

The observation of an anomalous relief of voltage-dependent block wherein neither equilibrium block nor the apparent off rate decline exponentially (as observed herein) is commonly viewed as evidence that the blocker has a finite, albeit modest, ability to transit the channel (Scheme II – from [66]). We have shown that this model offers an adequate description of HCN block by Mg^2+^ in the presence or absence of cAMP provided the bi-exponential behavior in the absence of agonist is interpreted through the lens of there being two populations of channels. This constraint is imposed because such a model predicts block will be inherently single exponential at any particular voltage and divalent ion concentration if it is kinetically decoupled from any linked process such as gating.

As noted earlier, there is an alternative explanation for anomalous relief of voltage dependent block, specifically that altered repulsion within a multi-ion pore can lead to the blocker being repelled back to the side from which it entered [67]. Accordingly, it is reasonable to ask whether analyzing our data within the formalism of that model would have altered the principal conclusion of our study that cAMP binding alters the open pore in a way that enhances the access of Mg^2+^ to its binding site. At the heart of the Heginbotham and Kutluay model is the idea that the Mg^2+^ site changes as a function of Mg^2+^ occupancy; to wit, the presence of the blocker changes ion loading in adjacent sites and alters the repulsive forces acting on the bound Mg^2+^ ion. That is, it postulates that channels are in one of two configurations. As such, it clearly allows for the emergence of bi-exponential block (the two sites are, by definition, different). However, the only way this model can explain the observation that one behavior predominates in the presence of cAMP but both contribute in the absence of ligand is to once again posit that gating ring activation leads to a propagated change that is sensed by ion binding sites in the pore. It would seem that such a consideration can be extended to any pore block model.

### The cyclic nucleotide dependence of intracellular Mg^2+^ block is evidence for loose coupling between the HCN channel gating ring and the pore

A number of studies have revealed that HCN channel gating is best reconciled with cyclic allosteric models (such as shown in Fig. 1B) wherein voltage-sensors can move irrespective of whether the pore is open or closed [30], [34], [35], [46], [47], [48], [49], [50], [51]. However, other findings suggest that such a model is inadequate. Thus, the extent of hysteresis under non-equilibrium conditions (such as in Fig. 9 and [51], [77]) and the sensitivity of tail current shape to activation strength [47], [51], [73], [77], [78] appear to be greater than anticipated within such a scheme while a reverse Cole-Moore effect is not explicable at all [57], [79]. Such findings have led to expansions of the model wherein the energetics of S4 motion are altered upon activation and/or opening [50], [51], [73] and coupling of the voltage sensors and activation gate can undergo a form of desensitization [57], [79]. In addition, it has been suggested there is at least one open state that lies off the activation path entirely [71].

However, none of these schemes can account for the cAMP-dependence of Mg^2+^ block observed here. Thus, in the basic concerted model and the voltage sensor “desensitized” model, all the open states are equivalent (although one could imagine that Mg^2+^ is sensing the different arrangements of the voltage sensors that lie at the heart of these models, this seems unlikely; see below). Similarly, while opening in different S4 modes and opening to the activation-decoupled arrangement can both incorporate distinct open states, S4 mode shifting is insensitive to cAMP while the agonist is reported to increase the probability of otherwise rare sojourns into the activation-decoupled open state [50], [51], [71], [73], [80].

**Figure 10:**
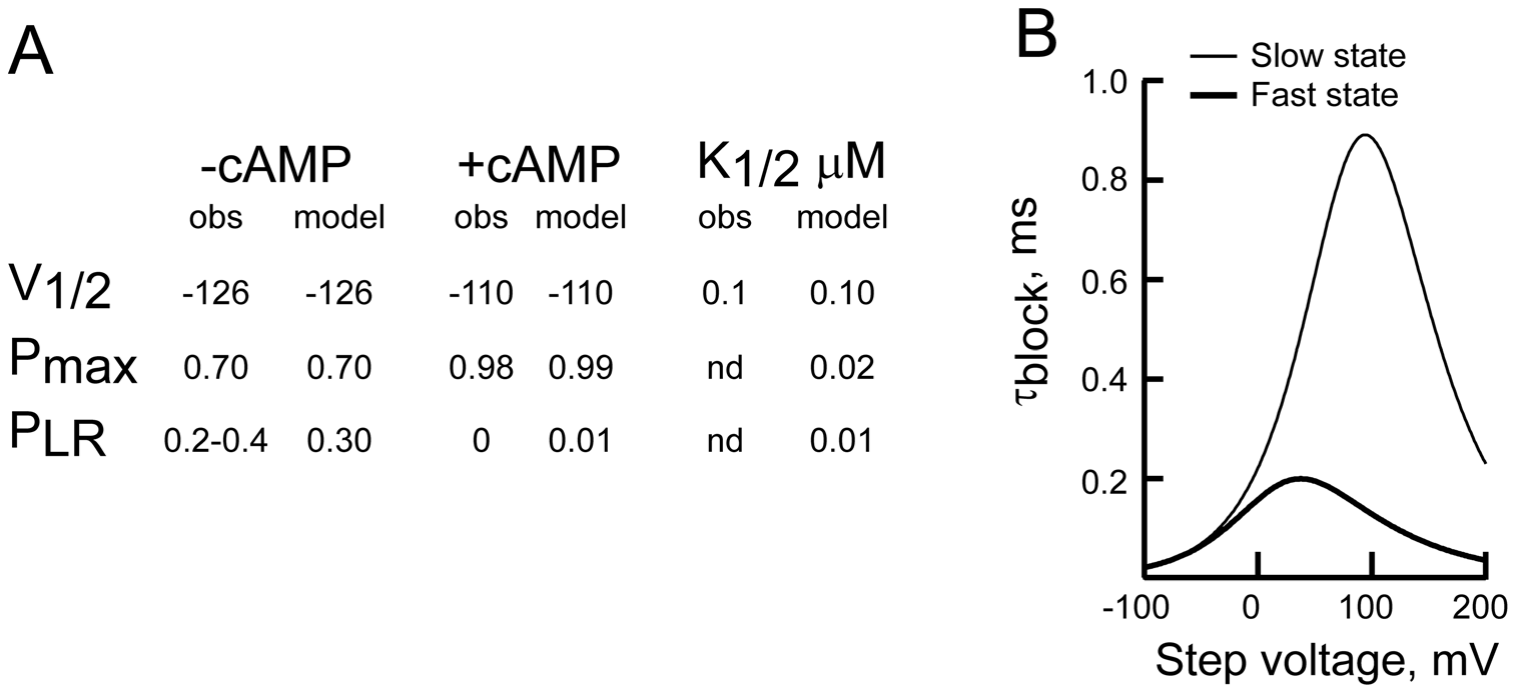
A modular model describes the cAMP enhancement of HCN2 activation and acceleration of [Mg^2+^]_in_ block. **A.** Observed (obs) and model generated (model) values of the V_1/2_ and P_MAX_ of channel activation and P_CL_ (the probability that the linker is in the resting configuration which we assume is reported as the slow component of block) each in the absence and presence of cAMP. The observed apparent affinities (K_1/2_) were either determined by fits of the Hill equation to model-generated concentration response curves or, for the observed K_1/2_ of cAMP modulation of gating, taken from published values [72]. **B.** Predicted behavior of τ_FAST_ (thick line) and τ_SLOW_ (thin line) as a function of the membrane potential. Curves were generated using equation 3 with k_2_ set to zero (see text for details).

An attractive alternative is offered from the work of Craven and Zagotta [30], [60]. To account for the influence of inter- and intra-subunit salt bridges within the cyclic nucleotide gating ring of HCN and CNG channels, these authors proposed that gating of HCN channels was better represented by a modular model (Fig. 1C) derived from that formulated by Horrigan and Aldrich to describe gating of the large conductance calcium-activated K^+^ channels [55]. This model is attractive because it explicitly partially decouples the activation status of components of the gating ring from the opening of the pore. Moreover, if we assume the resting configuration of the C-linker is synonymous with the slow blocking state (an idea considered further below), physiologically reasonable values of the equilibrium constants and allosteric factors can quantitatively describe both channel gating (V_1/2_ and P_MAX_ of opening in the absence and presence of cAMP as well as the apparent affinity for modification of the V_1/2_ by cAMP; [72]) and the presence of a slow blocking population in the absence, but not presence, of nucleotide (Fig. 10A).

### What could be the molecular origin of the slow blocking configuration?

There are two simple ideas we can consider: 1) There is some form of CNG channel-like propagated rearrangement wherein gating ring activation alters a barrier at, or above, the S6 bundle crossing. Such an effect of gating ring activation, wherein ion binding sites in the filter and vestibule are altered, is attractive because it is consistent with the effects of the gating ring observed in CNG channels and because there is an energetic coupling between the HCN channel selectivity filter and channel activation and/or opening ([81], [82], [83] and our unpublished observations). 2) The gating ring forms part of the permeation path itself and acts as the principal barrier for Mg^2+^ movement between the cytoplasm and its binding site. Thus, we can imagine that either the C-linker or the CNBD, when deactivated, act as part of the pore and restrict Mg^2+^ access. Clearly, the idea that this cytoplasmic extension of S6 can directly control Mg^2+^ block accords with the mechanism of polyvalent ion block observed in Kir channels [84], [85], [86]. One possible path through the HCN gating ring is represented by a negative charge-lined canal that lies directly below, and in line with, the S6 lined transmembrane pore. While mutagenesis experiments indicate that this path does not provide a significant barrier to permeant ions [87], such findings do not preclude the possibility that residues lining this canal could influence the progress of Mg^2+^.

Could different arrangements of the voltage sensors account for the cAMP sensitivity of block kinetics by influencing the electrostatic landscape the ions traverse? This seems unlikely because the amplitude of the slow block component is similar across a 100 mV range (compare the limiting values of A_f_/A_f_ + A_s_ between +100 mV and +200 mV in Figure 5F).

Finally, it is important to note that while the modular model shown in Figure 1C explicitly incorporates an activation step of the C-linker and we equate the effect of gating ring on the Mg^2+^ transition state as being due to the status of the C-linker, there is an implicit activation step for the CNBD as well but this is convolved with the ligand binding reaction for simplicity.

### Does loose coupling between the gating ring and pore help shape the response of HCN channels to pharmacological and natural product inhibitors?

A number of organic pore-block inhibitors of HCN channels have been described, including ivabradine (therapeutically marketed as Procoralan), nicotine, and ZD7288 (see [88] for review). While the nature of nicotine's high-affinity inhibition is presently based only on molecular modeling with respect to ZD7288 [89], observation suggests inhibition by ivabradine and ZD7288 depends on the architecture of the conduction path and/or the cAMP gating ring [2], [40], [90]. Thus, ivabradine-mediated inhibition exhibits a complex relationship to current flow [90], block by ZD7288 can have both reversible and irreversible components [2], and not only is the time course of block cAMP-sensitive [90] but ZD7288 binding perturbs association of the channel with cAMP [40].

Although superficially supportive of the hypothesis that loose coupling between the HCN pore and the gating ring may have a pharmacological correlate, the extant data do not allow this conclusion to be drawn. Thus, ivabradine binding has not been shown to have a cAMP-sensitivity while the coupling between nucleotide and ZD7288 can be readily explained within a strictly-coupled model. Unlike Mg^2+^, ZD7288 binds more tightly to closed HCN channels than open ones (albeit it can only access its site when the intracellular activation gate is open [2]). Given that cAMP biases the HCN opening reaction to the right while ZD7288 biases the same reaction to the left, thermodynamic interaction is to be expected though the interaction may yet be more complex [2], [40]. While these observations do not exclude a more nuanced basis for coupling between cAMP and the organic blockers, one that is predicated on the loose coupling described here, the slower kinetics of the larger inhibitors will make examination of this hypothesis difficult as an overlap with the kinetics of gating will make isolation of the blocking reaction problematic.

### Is there a chemical signaling role for divalent ion passage through HCN channels?

It has been suggested that alkaline earth metals are able to pass through the atypical HCN channel pore [74], [75], [76]. While our findings can be considered in terms of such a process, it is important to note that the magnitude of Mg^2+^ transport required to account for the anomalous off rate we observe would not represent a significant chemical flux. Does this suggest that the HCN pore has an unexpected selective permeability for Ca^2+^ over Mg^2+^? We think not. Rather, we suspect that the increase in intracellular Ca^2+^ concentration reported by Yu and colleagues [74], [75] arises from reversal of Na^+^-Ca^2+^ exchange in response to an HCN-mediated increase in internal Na^+^ while the Ca^2+^ permeable single channels analyzed by the Hoppe group [76], [91], [92] exhibit such atypical single channel and ensemble properties that assignment of this to HCN channels seems likely to be incorrect. Moreover, in light of the findings of Heginbotham and Kutluay [67], it is not even necessary to conclude that the anomalous relief we observe is necessarily a demonstration that Mg^2+^ passes through the channel at all.

### Physiological roles of cAMP dependent kinetics of Mg^2+^ block – implications with respect to the HCN subunit identity?

Figure 10B shows how the time constants of the fast and slow components of block will vary as a function of voltage at an “effectively physiological” Mg^2+^ concentration. For the simulation of the rapidly blocking component we used the values of 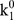, δ_1_, 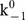 δ-_1_, 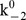, δ_−2_, determined in the presence of cAMP (see Table 1). To generate the behavior of the slow blocking component observed in the absence of cAMP, we used the values of 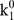 and δ_1_ determined for the slow component combined with the values 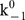, δ-_1_, 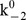 and δ_−2_, as used above. This approximation was both necessary (we do not have separate estimates of the off rates for the fast and slow components of block in the absence of cAMP) and reasonable (the off rates are less obviously altered by cAMP – see Figure 5B). For both solutions, we set the free Mg^2+^ concentration equal to 1.859 mM as it is only at this concentration that the equilibrium block in IOPC is as efficacious as is observed in intact cells [64]. It is apparent that cAMP unbound channels will block more slowly around or above action potential threshold than will cAMP bound channels. Such an observation suggests second messenger sensitivity of HCN channel rectification could contribute to control of a spike's shape and, in so doing, offer a novel way by which cellular excitability can be fine-tuned.

The gating properties of the four different HCN isoforms and heteromeric assemblies thereof show marked differences with respect to cAMP regulation of gating. Thus, HCN2 and 4 form channels whose slow (hundreds of milliseconds) and very slow (seconds) activation is strongly enhanced by cAMP [93], [94], [95], [96], [97], [98], HCN1 forms channels whose activation is relatively fast (tens to hundreds of milliseconds) but only modestly promoted by cAMP [97], [98], [99] while HCN3 channels have basal kinetics similar to HCN2 [98], [100], [101], [102] but they have the unusual property of being either insensitive to [101], or inhibited by, cAMP [102]. It will be interesting to determine whether Mg^2+^ block of each subunit, especially HCN3, tracks the cAMP-sensitivity of gating of the various isoforms or displays a distinct behavior. Given that the auxiliary protein, TRIP8b associates with the C-terminus of HCN channels and alters cAMP responsiveness [103], [104], [105], [106], the nature of cAMP-regulated Mg^2+^ block in the presence of this protein is of particular interest.

### Conclusion

The results presented here are consistent with the hypotheses that the gating ring of HCN channels is partially decoupled from channel opening as envisioned within a modular model and that the gating ring exerts a CNG channel-like propagated effect on the ion interaction landscape within the HCN channel pore. That this second messenger-mediated control of rectification may represent a novel expansion of the repertoire of cellular regulation exerted by the otherwise slow HCN channels is an intriguing possibility.

## Supporting Information

File S1 **Independence of the block time constant and current amplitude.** Single exponential block time constants, each relative to the mean value at the cognate Mg concentration and voltage, are plotted as a function of the amplitude of the inward current observed at −155 mV immediately prior to the block step. Data are from 63 independent patches recorded in the presence of cAMP. Lines represent linear regressions to the data obtained at 50, 100, 150 and 200 mV according to the gray scale indicated in the legend. The R^2^ values for each regression were 0.0038, 0.0402, 0.0228 and 0.0255.
(PDF)

File S2 **Derivations of equations describing block models.**
(PDF)

File S3 **HCN2 single channel conductance-voltage properties as determined by non-stationary fluctuation analysis.** **A.** Representative plot of 300 consecutive outward HCN2 tail currents obtained in the absence of internal Mg and the presence of 30 µM cAMP (red traces). The activating voltage step was 1 s at −155 mV. Tails were recorded at +40 mV. The inter-pulse interval was 8 s. Records were filtered at 10 kHz and sampled at 50 kHz. Residual leak current not eliminated by analogue circuitry was subtracted from each record before display here or analysis for NSFA. The black trace is the mean of these records. **B.** The variance (obtained from 0.5 times the mean of the squared difference between sequential pairs of sweeps [68], [69]) of the final ∼10% of the deactivating records is plotted as a function of current amplitude. The superimposed straight line fit yields a single channel current of 95 fA corresponding to a single channel conductance of 2.4 pS. The background variance (1.48×10^−24^ A^2^) has been subtracted from the raw data and fit line for clarity. From a number of such recordings, the mean single channel conductance of HCN2 was determined to be 2.1 pS±0.4, n = 5 and 2.7 pS±0.6, n = 4 in the presence and absence of cAMP respectfully. As these values are not statistically different, we use an average value of 2.4 pS in all calculations. In doing so, we assume that the outward single channel IV relationship is linear. This seems reasonable given that measures of the single channel conductance at hyperpolarized potentials are similar to the above values. Thus, we find that at −155 mV the single channel conductance is 2.1 pS±0.2, n = 9 and 2.3 pS±0.3, n = 5 in the presence and absence of cAMP respectfully, values that are in close agreement with reported values of 1.5 to 2.9 pS for HCN2 at hyperpolarized potentials [87], [107].
(PDF)
