# Supplementary figures and images for: cAMP Control of HCN2 Channel Mg2+ Block Reveals Loose Coupling between the Cyclic Nucleotide-Gating Ring and the Pore
Source: PLoS One. 2014 Jul 1;9(7):e101236. doi: 10.1371/journal.pone.0101236 (PMC4077740; doi:10.1371/journal.pone.0101236)

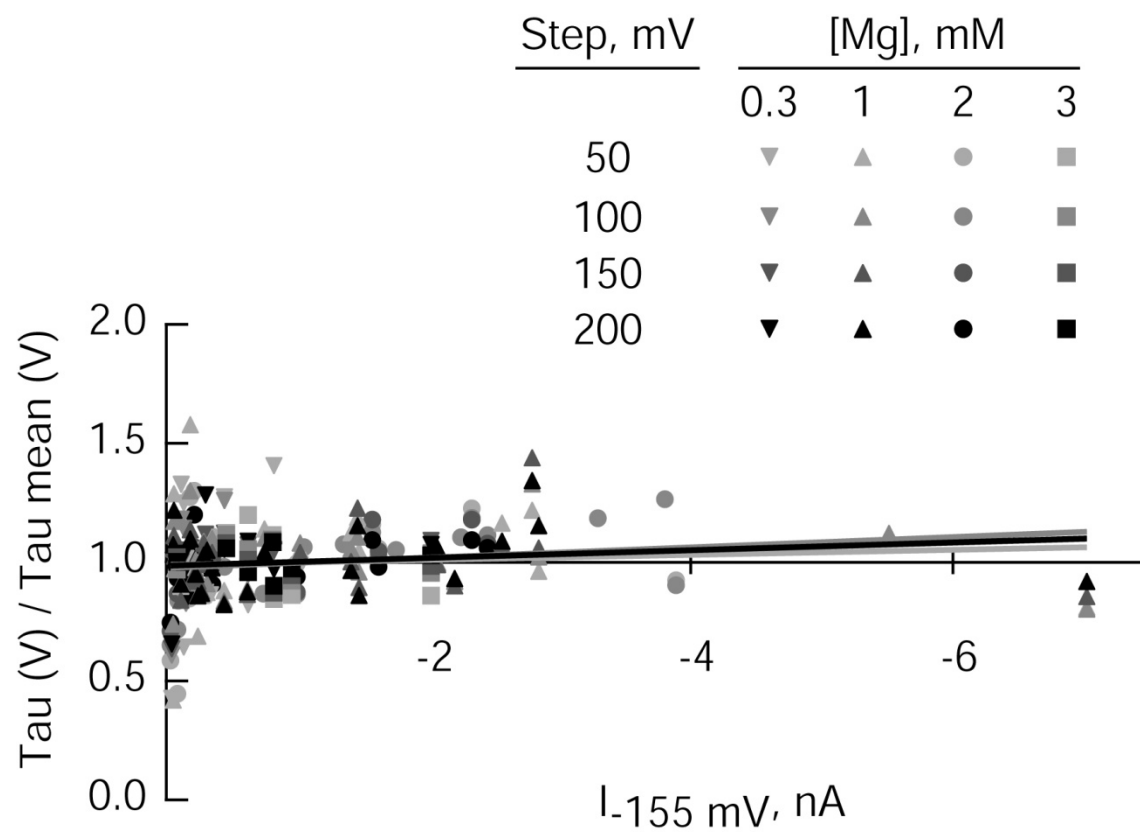

**Supplemental File S1 - Independence of the block time constant and current amplitude.**

Supplement: File S1 — Independence of the block time constant and current amplitude. Single exponential block time constants, each relative to the mean value at the cognate Mg concentration and voltage, are plotted as a function of the amplitude of the inward current observed at −155 mV immediately prior to the block step. Data are from 63 independent patches recorded in the presence of cAMP. Lines represent linear regressions to the data obtained at 50, 100, 150 and 200 mV according to the gray scale indicated in the legend. The R2 values for each regression were 0.0038, 0.0402, 0.0228 and 0.0255. (PDF) [file pone.0101236.s001.pdf]
