# Supplementary material for: cAMP Control of HCN2 Channel Mg2+ Block Reveals Loose Coupling between the Cyclic Nucleotide-Gating Ring and the Pore
Source: PLoS One. 2014 Jul 1;9(7):e101236. doi: 10.1371/journal.pone.0101236 (PMC4077740; doi:10.1371/journal.pone.0101236)
Supplement: File S2 — Derivations of equations describing block models. (PDF) [file pone.0101236.s002.pdf]

Supplemental File S2 - Derivations of equations describing block models

Block Schemes

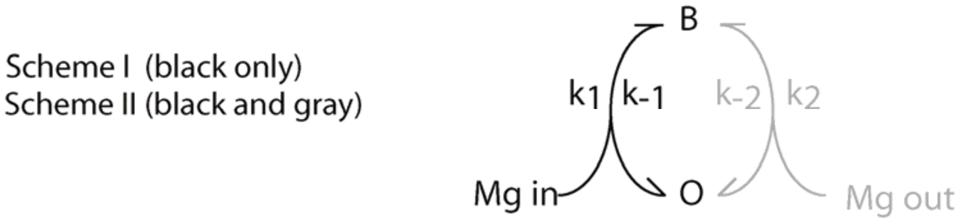

Analysis of kinetics of permeant block (scheme II)

Scheme II can be described by $$dO/dt = -k_1.[Mg]_{in}.O_t - k_2.[Mg]_{out}.O_t + k_{-1}.B_t + k_{-2}.B_t \quad (S1)$$

Let $$a = k_1.[Mg]_{in}$$

$$b = k_2.[Mg]_{out}$$

$$c = k_{-1}$$

$$d = k_{-2}$$

So $$dO/dt = -a.O_t - b.O_t + c.B_t + d.B_t \qquad (S2)$$

As $$B_t = 1 - O_t$$

$$dO/dt = -a.O_t - b.O_t + c.(1-O_t) + d.(1-O_t)$$

$$dO/dt = -a.O_t - b.O_t + c - c.O_t + d - d.O_t$$

Then $$dO/dt = (c+d) - (a+b+c+d).O_t \qquad (S3)$$

At equilibrium $$dO/dt = 0 = (c+d) - (a+b+c+d).O_\infty$$

So $$(c+d) = (a+b+c+d).O_\infty \qquad (S4)$$

Substitution of S4 into S3 yields $$dO/dt = (a+b+c+d).O_\infty - (a+b+c+d).O_t$$

Which rearranged gives $$dO/dt = (a+b+c+d).(O_\infty - O_t) \qquad (S5)$$

When S5 is multiplied by -1 $$-dO/dt = (a+b+c+d).(O_t - O_\infty)$$

Then rearranged $$dO/(O_t - O_\infty) = -(a+b+c+d).dt$$

And integrated, we get $$Ln|(O_t - O_\infty)| = -(a+b+c+d).t + K \qquad (S6)$$

At t=0 equation S6 gives $$\mathrm{Ln}\left|(O_0 - O_\infty)\right| = K \quad \text{(S7)}$$

Substitution of S7 into S6 yields $$\mathrm{Ln}\left|(O_t - O_\infty)\right| = -(a+b+c+d).t + \mathrm{Ln}\left|(O_0 - O_\infty)\right|$$

Or $$\mathrm{Ln}\frac{\left|(O_t - O_\infty)\right|}{\left|(O_0 - O_\infty)\right|} = -(a+b+c+d).t$$

Taking exponents $$\frac{\left|(O_t - O_\infty)\right|}{\left|(O_0 - O_\infty)\right|} = e^{-t/\tau}$$

And rearranging gives $$O_t = O_\infty + (O_0 - O_\infty)e^{-t/\tau} \quad \text{(S8)}$$

Where $$\tau^{-1} = a + b + c + d \quad \text{(S9)}$$

Thus $$\tau^{-1} = k_1.[\mathrm{Mg}]_{in} + k_2.[\mathrm{Mg}]_{out} + k_{-1} + k_{-2} \quad \text{(S10)}$$

As, under our conditions, the only variables are $[\mathrm{Mg}]_{in}$ and voltage, V (wherein V appears in the four rate constants according to equations S11-S14), equation 10 shows that τ^{-1} should be linearly related to $[Mg^{2+}]_{in}$ and that the regression lines of such plots will have slopes equal to k_1 and intercepts on the ordinate equal to $k_2.[\mathrm{Mg}]_{out} + k_{-1} + k_{-2}$ (see Figure 3).

$$k_1 = k_1^0.\ e^{zFV\delta_1/RT} \quad \text{(S11)}$$

$$k_{-1} = k_{-1}^0.\ e^{-zFV\delta_{-1}/RT} \quad \text{(S12)}$$

$$k_2 = k_2^0.\ e^{-zFV\delta_2/RT} \quad \text{(S13)}$$

$$k_{-2} = k_{-2}^0.\ e^{zFV\delta_{-2}/RT} \quad \text{(S14)}$$

Where $$\delta = \delta_1 + \delta_{-1}$$

And $$1 - \delta = \delta_2 + \delta_{-2}$$

Importantly, this examination shows that the introduction of permeant block is not alone sufficient to account for the observed bi-exponential behavior of the block; block at any unique combination of voltage and $[Mg^{2+}]$ is determined by a simple, and single, value of τ. Note that equation S8 is identical to the function used in our single component exponential fitting routine.

Analysis of Probability of unblock, P_{UN}, within scheme II

$$P_{UN} = \frac{O}{O+B} = \frac{1}{1+\frac{B}{O}} \qquad \text{(S15)}$$

At equilibrium when $d0/dt = 0$

$O = O_\infty$

$B = B_\infty$

We get, from equation, S1 $k_1.[Mg]_{in}.O_\infty + k_2.[Mg]_{out}.O_\infty = k_{-1}.B_\infty + k_{-2}.B_\infty$

Which, rearranged yields $O_\infty\left(k_1.[Mg]_{in} + k_2.[Mg]_{out}\right) = B_\infty(k_{-1} + k_{-2})$

And hence

$$\frac{B_\infty}{O_\infty} = \left(k_1.[Mg]_{in} + k_2.[Mg]_{out}\right)/(k_{-1} + k_{-2}) \qquad \text{(S16)}$$

Substituting S16 into S15 yields

$$P_{UN} = (k_{-1} + k_{-2}) \ / \ (k_1.[Mg]_{in} + k_2.[Mg]_{out} + k_{-1} + k_{-2}) \qquad \text{(S17)}$$

Which can be rewritten as

$$P_{UN} = (k_{-1} + k_{-2}) * \tau \qquad \text{(S18)}$$

Equation S17 is equivalent to Woodhull's equation 1a [1]. Equation S17 can also be readily obtained if we start from the mean block and mean unblock times (as per equations S19 and S20).

$$\text{Mean block time} = 1/(k_{-1} + k_{-2}) \qquad \text{(S19)}$$

$$\text{Mean unblock time} = 1/(k_1.[Mg]_{in} + k_2[Mg]_{out}) \qquad \text{(S20)}$$

Comparison of Scheme II equations to those describing impermeant block (Scheme I)

Setting k_2 and k_{-2} to zero (abolishing access of external Mg^{2+} to the block site) yields equations identical to those derived for impermeant block (as expected).

$$\frac{|(O_t - O_\infty)|}{|(O_0 - O_\infty)|} = e^{-t/\tau} \qquad \text{Where} \qquad \tau^{-1} = k_1.[Mg]_{in} + k_{-1}$$

$$P_{UN} = 1/\left(1 + (k_1.[Mg]_{in}/k_{-1}\right)$$

References

1. Woodhull AM (1973) Ionic blockage of sodium channels in nerve. J Gen Physiol 61: 687-708.
