# Supplementary material for: cAMP Control of HCN2 Channel Mg2+ Block Reveals Loose Coupling between the Cyclic Nucleotide-Gating Ring and the Pore
Source: PLoS One. 2014 Jul 1;9(7):e101236. doi: 10.1371/journal.pone.0101236 (PMC4077740; doi:10.1371/journal.pone.0101236)
Supplement: File S3 — HCN2 single channel conductance-voltage properties as determined by non-stationary fluctuation analysis. A. Representative plot of 300 consecutive outward HCN2 tail currents obtained in the absence of internal Mg and the presence of 30 µM cAMP (red traces). The activating voltage step was 1 s at −155 mV. Tails were recorded at +40 mV. The inter-pulse interval was 8 s. Records were filtered at 10 kHz and sampled at 50 kHz. Residual leak current not eliminated by analogue circuitry was subtracted from each record before display here or analysis for NSFA. The black trace is the mean of these records. B. The variance (obtained from 0.5 times the mean of the squared difference between sequential pairs of sweeps [68], [69]) of the final ∼10% of the deactivating records is plotted as a function of current amplitude. The superimposed straight line fit yields a single channel current of 95 fA corresponding to a single channel conductance of 2.4 pS. The background variance (1.48×10^−24^ A2) has been subtracted from the raw data and fit line for clarity. From a number of such recordings, the mean single channel conductance of HCN2 was determined to be 2.1 pS±0.4, n = 5 and 2.7 pS±0.6, n = 4 in the presence and absence of cAMP respectfully. As these values are not statistically different, we use an average value of 2.4 pS in all calculations. In doing so, we assume that the outward single channel IV relationship is linear. This seems reasonable given that measures of the single channel conductance at hyperpolarized potentials are similar to the above values. Thus, we find that at −155 mV the single channel conductance is 2.1 pS±0.2, n = 9 and 2.3 pS±0.3, n = 5 in the presence and absence of cAMP respectfully, values that are in close agreement with reported values of 1.5 to 2.9 pS for HCN2 at hyperpolarized potentials [87], [107]. (PDF) [file pone.0101236.s003.pdf]

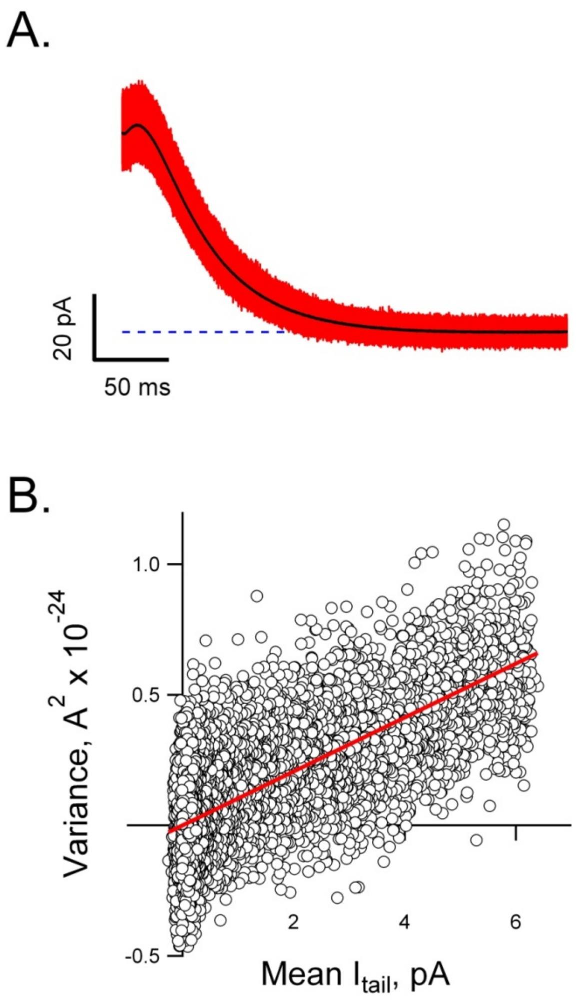

Supplemental File S3 - HCN2 single channel conductance-voltage properties as determined by non-stationary fluctuation analysis.
